# Dysfunction in the arbuscular mycorrhizal symbiosis has consistent but small effects on the establishment of the fungal microbiota in *Lotus japonicus*

**DOI:** 10.1111/nph.15958

**Published:** 2019-07-02

**Authors:** Li Xue, Juliana Almario, Izabela Fabiańska, Georgios Saridis, Marcel Bucher

**Affiliations:** ^1^ Botanical Institute Cologne Biocenter University of Cologne 50674 Cologne Germany; ^2^ Cluster of Excellence on Plant Sciences (CEPLAS) University of Cologne 50674 Cologne Germany

**Keywords:** arbuscular mycorrhizal (AM) fungi, fungal community, legume, natural soil, RNA‐seq, symbiosis

## Abstract

- Most land plants establish mutualistic interactions with arbuscular mycorrhizal (AM) fungi. Intracellular accommodation of AM fungal symbionts remodels important host traits like root morphology and nutrient acquisition. How mycorrhizal colonization impacts plant microbiota is unclear.
- To understand the impact of AM symbiosis on fungal microbiota, ten *Lotus japonicus* mutants impaired at different stages of AM formation were grown in non‐sterile natural soil and their root‐associated fungal communities were studied.
- Plant mutants lacking the capacity to form mature arbuscules (arb^−^) exhibited limited growth performance associated with altered phosphorus (P) acquisition and reduction–oxidation (redox) processes. Furthermore, arb^−^ plants assembled moderately but consistently different root‐associated fungal microbiota, characterized by the depletion of Glomeromycota and the concomitant enrichment of Ascomycota, including *Dactylonectria torresensis*. Single and co‐inoculation experiments showed a strong reduction of root colonization by *D. torresensis* in the presence of AM fungus *Rhizophagus irregularis*, particularly in arbuscule‐forming plants.
- Our results suggest that impairment of central symbiotic functions in AM host plants leads to specific changes in root microbiomes and in tripartite interactions between the host plant, AM and non‐AM fungi. This lays the foundation for mechanistic studies on microbe–microbe and microbe–host interactions in AM symbiosis of the model *L. japonicus*.

## Introduction

Understanding how microbial communities assemble in plant roots from a soil species pool is a central issue in microbial ecology and could affect modern breeding of climate‐resilient crops with their associated microbiota (Dumbrell *et al*., 2010; Orrock & Watling, 2010). A few symbiotic associations of land plants with soil microbes have been extensively studied mechanistically, including root nodule (RN) symbiosis with Rhizobia and *Frankia* bacteria in leguminous and actinorhizal plants, and the arbuscular mycorrhizal (AM) symbiosis with soil fungi from the phylum Glomeromycota in most land plants (Benson & Silvester, 1993; Parniske, 2008; Oldroyd *et al*., 2011). In AM symbiosis, the fungal partner scavenges mainly phosphorus (P) from the soil and transfers it to the host plant in exchange for photosynthetic carbon in the form of sugars and lipids (Shachar‐Hill *et al*., 1995; Bago *et al*., 2000; Bravo *et al*., 2017; Jiang *et al*., 2017; Keymer *et al*., 2017; Luginbuehl *et al*., 2017). The bidirectional nutrient exchange between land plants and Glomeromycota fungi is thought to have stabilized the mutualistic interaction during ~ 400 Myr of coevolution (Remy *et al*., 1994; Kiers *et al*., 2011). Symbiotic nutrient exchange takes place at the interface between specialized fungal tree‐like cellular structures, so called arbuscules, and the host root. Arbuscules undergo developmental processes from intracellular trunk formation through hyphal fine‐branching to degeneration (Gutjahr & Parniske, 2013). The accommodation of AM fungi within root cells is coordinated by a complex network of signals and plant proteins. Receptor‐like kinase SYMRK/DMI2 is involved in the sensing and transduction of the fungal signal (Endre *et al*., 2002; Stracke *et al*., 2002), which triggers calcium spiking in the plant nucleus. The calcium–calmodulin‐dependent protein kinase CCaMK/DMI3 decodes the calcium signal and mediates subsequent transcriptional regulation in the nucleus (Lévy *et al*., 2004). Forming a protein complex with CCaMK and DELLA protein, CYCLOPS/IPD3 acts as a transcriptional activator and directly regulates expression of the *RAM1* gene (Pimprikar *et al*., 2016), encoding a GRAS transcription factor which regulates *RAM2* (Gobbato *et al*., 2012). The *RAM2* gene encodes a glycero‐3‐phosphate acyl transferase (Wang *et al*., 2012) required for the synthesis of 16:0 β‐monoacylglycerols (β‐MAG) (Bravo *et al*., 2017; Jiang *et al*., 2017; Keymer *et al*., 2017; Luginbuehl *et al*., 2017). AP2 transcription factors CBX1 and WRI5a co‐regulate a divergent set of genes underlying mycorrhizal phosphate uptake and lipid biosynthesis (Jiang *et al*., 2018; Xue *et al*., 2018). β‐MAGs are subsequently exported to the fungus probably via half‐ABC transporters STR to maintain the symbiotic relationship (Bravo *et al*., 2017; Jiang *et al*., 2017; Keymer *et al*., 2017; Luginbuehl *et al*., 2017).

SYMRK, CYCLOPS and CCaMK are also engaged in root nodule (RN) symbiosis with nitrogen‐fixing rhizobia as components of the ‘common symbiosis signaling pathway’ (CSSP) (Kistner & Parniske, 2002). It has been suggested that some of the AM symbiosis genes have been co‐opted by plant pathogens. RAM2 and the GRAS protein RAD1 are required for the full colonization both by AM fungi and by *Phytophthora palmivora* (Wang *et al*., 2012; Rey *et al*., 2015, 2017; Xue *et al*., 2015). On the other hand, some CSSP genes play dual roles in AM symbiosis and plant defense as shown by the knockdown of tomato *CCaMK* leading to reduced resistance against *Sclerotinia sclerotiorum* and *Pseudomonas syringae* pv*. tomato* DC3000 (Wang *et al*., 2015). Similarly, the rice LysM receptor‐like kinase CERK1 is required both for AM symbiosis establishment and for resistance against blast fungus *Magnaporthe oryzae* (Zhang *et al*., 2015). Beyond these binary interaction studies, it is currently unknown whether AM symbiosis‐related genes are essential for root colonization by other plant‐associated microbes and, more generally, whether these genes are involved in shaping of entire root‐associated microbial communities in natural soil.

It is known that AM symbiosis establishment triggers local and systemic changes in the host′s root system architecture (Hetrick, 1991; Gutjahr *et al*., 2009; Paszkowski & Gutjahr, 2013), root exudation (Mada & Bagyaraj, 1993; Bansal & Mukerji, 1994) and plant defense (Jung *et al*., 2012). Mycorrhizal plants exhibit increased resistance to root pathogens (Azcón‐Aguilar & Barea, 1997; Cameron *et al*., 2013). Underlying mechanisms likely involve activation of plant defense by AM fungi (Cordier *et al*., 1998; Pozo *et al*., 2002) and inhibition of the pathogen by antagonism or competition for resources (Green *et al*., 1999). Additionally, AM fungi are thought to scavenge reactive oxygen species (ROS) so as to counteract the stress responses and enhance plant tolerance (Nath *et al*., 2016). Cellular ROS present at low levels are considered to act as signaling molecules in physiological functions, while high amounts of ROS are related to oxidative stress (Foyer & Noctor, 2005; Mittler, 2017). The transferability of these observations to the situation in natural ecosystems is still unclear.

In this study, we sought to determine the impact of AM symbiosis dysfunction on plant growth and on the assembly of root microbiota in natural soil. Our first aim was to test whether root‐associated fungal communities respond to the genetic perturbation of AM formation. Using amplicon sequencing of the fungal taxonomic marker ITS2, we compared root‐associated fungal communities of ten *Lotus japonicus* mutant lines impaired at different stages of AM establishment and showed an effect of the AM symbiosis on the formation of the root microbiota. Our second aim was to reconstitute the observed community shifts in a gnotobiotic system in order to understand causality effects. The Ascomycota fungus *Dactylonectria torresensis* originating from *L. japonicus* roots was thus co‐inoculated with AM fungus *Rhizophagus irregularis* in a gnotobiotic system to explore the displacement of this non‐AM fungal member of the microbiota by the AM fungus in normal mycorrhizal roots.

## Materials and Methods

### Plant growing conditions and fungal colonization analyses

The *L. japonicus* genotypes used in this study are in the Gifu B‐129 (WT) background (Supporting Information Table S1). Seeds of wild type (WT), *symrk‐10*,* cyclops‐3*,* ram1‐1* (30002740), *ram1‐2* (30082472), *ram2‐2* (30000742), *ram2‐3* (30002873)*, rad1‐2* (30030576), *rad1‐3* (30052260), *str‐1* (30001288) and *str‐2* (30055073), were surface‐sterilized and germinated on 0.8% agar plates for 7 d before transplanting in sand–soil gnotobiotic systems or natural soil.

For experiments in the sand/soil gnotobiotic systems, seedlings were transferred to pots filled with 0.5 l of sterile sand/soil mixture (9 : 1) with or without *R. irregularis* inoculum (BEG74). Plants grew in a walk‐in‐chamber (16 h : 8 h, light : dark, 24 : 18°C, 70 : 55% humidity) and were fertilized with half‐strength Hoagland's solution (including 10 μM NH_4_H_2_PO_4_) once a week. After 6 wk, plants were harvested.

For the experiments in natural soil, seedlings were transferred to pots filled with 1 l NPK soil (Table S2) (Willmann *et al*., 2013; Almario *et al*., 2017). Plants were grown as described above, pots were randomized and watered every other day to keep soil humidity at 70% of the water retention capacity. After 6 wk, five to six plants per pot were harvested and pooled. The combined roots were cut and homogenized before subsampling. Root samples were taken to (1) score fungal colonization via microscopy after staining, (2) extract RNA for gene expression analysis, and (3) collect root and rhizosphere fractions for fungal community analysis (described below). Shoots were weighed and used for P analysis. The experiment was performed twice with all plant lines (Expts 1 and 2) and additionally once with WT, *symrk‐10*,* ram2‐2* and *ram2‐3* (Expt 3) with five to six pots per genotype (Table S3). Three unplanted pots with bulk soil (BS) were included in Expts 1 and 2.

The *in planta* interaction between AM fungus *R. irregularis* and *D. torresensis* was studied in sand/soil gnotobiotic systems. WT, *symrk‐10* and *cyclops‐3* were planted with or without *R. irregularis* inoculum as described above. One day after transplanting, selected plants were inoculated with *D. torresensis* isolate 107 (deposited in in‐house collection of indexed fungal isolates) which exhibited 100% sequence identity with the ITS2 sequence representative of *D. torresensis* OTU00003. For *D. torresensis* 107 inoculation, MYP agar plates (7 g malt extract, 1 g tryptone‐peptone, 0.5 g yeast extract and 12 g agar l^‐1^) were used to prepare fungal inoculation suspensions (Almario *et al*., 2017). Briefly, fungal mycelium was weighed, diluted in 1 ml sterile water and ground with glass beads in a Percellys 24 (Bertin Instruments; twice for 10 s at 6200 rpm). The fungal suspension was washed twice with sterile water and diluted to 10 mg ml^−1^. After growing the plants for 6 wk, plant shoots were weighed, and roots were washed in deionized water, blot dried and frozen in liquid nitrogen before DNA extraction. Extracted DNA was further used to quantify fungal colonization using qPCR. *Thelonectria olida* isolates 57 and 102, phylogenetically distant from *D. torresensis* OTU00003 (<90% ITS2 sequence similarity) but also belonging to the Nectriaceae family (based on their ITS2 sequence), were inoculated in the same way.

### Root and rhizosphere sampling and ITS2 sequencing data analysis

Rhizosphere and root samples were collected using a fractionation method described previously (Almario *et al*., 2017). The obtained paired‐end reads were processed in mothur v1.37.3 (Schloss *et al*., 2009) using a custom pipeline and the UNITE fungal ITS database (v.7.2, release 1.12.2017) (Kõljalg *et al*., 2013). The raw ITS2 sequencing data are deposited at NCBI sequence read archive under Bioproject PRJNA489990.

### RNA extraction and qRT‐PCR

The RNA extraction and qRT‐PCR were performed as described previously (Xue *et al*., 2018). All primers used are listed in Table S4.

### RNA‐seq data analysis

Datasets of the short reads have been deposited at NCBI under accession number PRJNA518045. RNA‐seq analysis was performed as described previously (Xue *et al*., 2018). Genes with log_2_ fold change ≥ 2 or ≤ −2 (adjusted *P* ≤ 0.05) were identified as differentially expressed genes (DEG) (Tables S5, S6). In order to draw the heatmaps, values of log_10_ (counts per million (CPM) + 1) were used. The CPM values were calculated by edgeR after normalizing for library sizes. To retrieve the overrepresented gene ontology (GO) terms, agriGO was used (Fisher's test with Hochberg‐FDR correction) (Du *et al*., 2010), using a customized annotated reference, and the ‐log_10_ transformed *P*‐values were used to draw the heatmap. Re‐analysis of published datasets was used to establish reference gene sets: inoculated vs non‐inoculated roots fertilized with low Pi for *R. irregularis‐*regulated genes (Table S7) (Handa *et al*., 2015) and WT fertilized with low Pi (5 μM NH_4_H_2_PO_4_) vs high Pi (7.5 mM NH_4_H_2_PO_4_) for phosphate starvation response (PSR) genes (Table S8) (Xue *et al*., 2018).

### Quantitative real‐time PCR of *R. irregularis* and *D. torresensis in planta*

The DNA of *R. irregularis* and *D. torresensis* was quantified by real‐time PCR. Real‐time PCR reactions were conducted in 20 μl containing 2 μl DNA (4 ng μl^−1^), 10 μl SYBR green master mix (Applied Biosystems, Birchwood, UK) and 10 pM of each primer. A QuantStudio^™^ 5 System (Thermo Fisher, Waltham, MA, USA) was used and primers and cycling conditions were adapted for each fungus. The efficiencies of the real‐time PCR quantification methods were analyzed using varying amounts of fungal DNA (0.01, 0.1 and 1.0 ng).

Around 300 spores of *R. irregularis* (BEG74) were purified from open pot cultures planted with chives for 5 months, by using wet‐sieving and sucrose‐gradient centrifugation (http://www.i-beg.eu/protocols.htm). The pure genomic DNA extracted from the spores was serially diluted and quantified with primers gRiLSU‐F/gRiLSU‐R and medium‐stringency cycling conditions (95°C for 15 min; 45 cycles: 95°C for 10 s, 54°C for 20 s, 72°C for 5 s) as described (Thonar *et al*., 2012).

After harvesting from plates, fungal isolates were grown on MYP medium and pure genomic DNA was extracted as described (Cenis, 1992). *Dactylonectria torresensis* 107 DNA was quantified with primers YT2F/Cyl_R and cycling conditions (95°C for 10 min, 60 cycles: 95°C for 10 s, 60°C for 10 s, 72°C for 30 s) as described (Agustí‐Brisach *et al*., 2014). DNA of *T. olida* isolates 57 and 102 was quantified with primer pair YT2F/102_Cyl_R using the same cycling conditions.

For *in planta* fungal DNA quantification, total DNA was extracted from root samples using the FastDNA^™^ SPIN Kit. Relative amounts of fungal DNA to plant DNA were calculated using the 2^−ΔCt^ method and the *L. japonicus Ubiquitin* gene as reference, or the 2^−ΔΔCt^ method (Livak & Schmittgen, 2001) using *L. japonicus Ubiquitin* as reference gene and ‘Single inoculation GifuB‐129’ as reference treatment. Fungus‐specific primers are listed in Table S4.

### Statistical analyses

The Rstudio (v.3.2.1) was used for statistical analyses (RStudio, 2015). The operational taxonomic unit (OTU) table was used to quantify OTU relative abundances, which were log_10_(*X* + 1) transformed. This final transformed OTU table was used to calculate Bray‐Curtis dissimilarities between samples using the ‘vegdist’ function of the vegan package (Oksanen *et al*., 2016). The ‘dudi.pco’ and ‘s.class’ functions from the ade4 package were used to conduct the principal coordinates analyses (PCoA). Permutational multivariate analysis of variance (PerMANOVA) on Bray‐Curtis dissimilarities was conducted using the ‘adonis’ function of the vegan package (at *P* < 0.05, 10 000 permutations; Tables S9, S10).

Taxa showing differences in their relative abundance between samples from arb^+^ and arb^−^ plants were identified using the DESeq function in the DESeq2 package (*P* < 0.05, FDR correction with the Benjamini–Hochberg procedure) (Love *et al*., 2014). The raw counts were normalized with ‘estimateSizeFactors’ function and modeled using a negative binomial generalized linear model. The average relative abundances of these OTUs were used to generate a heatmap using hierarchical clustering (with one minus Pearson's correlation and average linkage). Relative abundances of the identified fungal OTUs in arb^+^ and arb^−^ plants were further compared with Wilcoxon test (FDR corrected *P* < 0.05; Table S11). Unless otherwise stated, means were compared using one‐way ANOVA followed by Tukey's HSD test (*P* < 0.05), Kruskal‐Wallis test followed by Dunn's test (with Benjamini–Hochberg correction; *P* < 0.05) or Wilcoxon's test (*P* < 0.05).

The RNA extraction and qRT‐PCR, sampling and ITS2 sequencing, recovery of root fungal isolates, ICP‐MS, scoring of AM fungal colonization and Gene ID are described in Methods S1.

## Results

### Mycorrhizal phenotypic variation defines growth vigor in *L. japonicus* in natural soil

**Figure 1:**
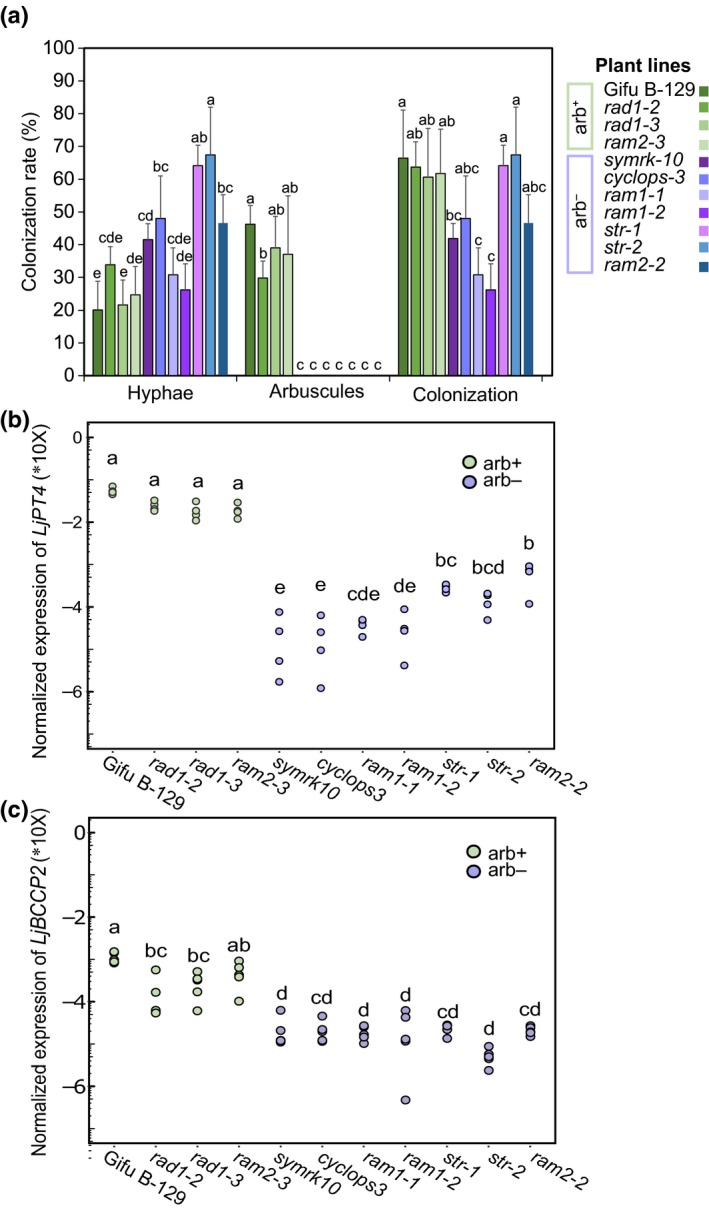
Fungal colonization and *LjPT4* expression in the roots of *Lotus japonicus* lines growing in an agricultural (NPK) soil for 6 wk. (a) Percentage of plant roots with observable fungal colonization (Hyphae) or with well‐developed arbuscules (Arbuscules). ‘Colonization’ indicates the sum of ‘Hyphae’ plus ‘Arbuscules’ representing the overall percentage of plant roots colonized by fungal structures. No arbuscules or aberrant arbuscules were observed in arb^−^ lines. Error bars represent + SD (*n* = 5). (b) and (c) Root expression of mycorrhiza‐induced phosphate transporter gene *LjPT4* and fatty acid biosynthesis gene *LjBCCP2*, respectively. In both panels different letters designate significant differences between the plant lines (ANOVA followed by Tukey's HSD test, *P *<* *0.05, *n* = 4 or 5). Both analyses were conducted in three independent experiments with similar results. Results from Expt 1 are shown.

Ten mutant lines defective in AM symbiosis genes (Table S1; Fig. S1) were grown in a simplified substrate containing *R. irregularis* inoculum. Lines *symrk‐10*,* cyclops‐3*,* ram1‐1*,* ram1‐2* and *ram2‐2* showed no arbuscule formation or only degenerated arbuscules (phenotype designated as arb^−^) accompanied by strongly reduced expression of *LjPT4*, a plant marker gene indicating functional AM symbiosis (Gutjahr & Parniske, 2013), while lines *rad1‐2* and *rad1‐3* showed a WT‐like AM fungal colonization level (arb^+^) with reduced levels of *LjPT4* transcripts (*t*‐test, *P* < 0.05) (Fig. S2a–c). Two as yet unpublished lines *str‐1* and *str‐2* displayed a stunted arbuscule phenotype, as the *str* allele in MG‐20 background (Keymer *et al*., 2017). The *ram2‐3* mutant showed an arb^+^ phenotype (Fig. S2a–c). All the plant lines had similar P content (Fig. S2d) and shoot fresh weight (Fig. S2e). To assess the contribution of CSSP and downstream genetic factors to the growth performance in natural soil, these mutant lines and WT were grown on an agricultural soil (‘NPK soil’; Table S2). Arbuscule formation (Fig. 1a) and expression of marker genes *LjPT4* and *LjBCCP2* encoding biotin carboxyl carrier protein 2 (Fig. 1b,c) were similar to those observed in the simplified substrate. In contrast to binary conditions (Fig. S2e), arb^−^ plants exhibited significantly reduced shoot growth (Fig. S3a). Similarly, shoot element profiles, and particularly P, K and S content, differed significantly between arb^+^ and arb^−^ genotypes (Fig. S3b), suggesting that specifically in natural soil aberrant arbuscule development strongly affected plant growth and acquisition of nutrients.

### Mutants defective in AM symbiosis exhibit distinctive transcriptomes in natural soil

**Figure 2:**
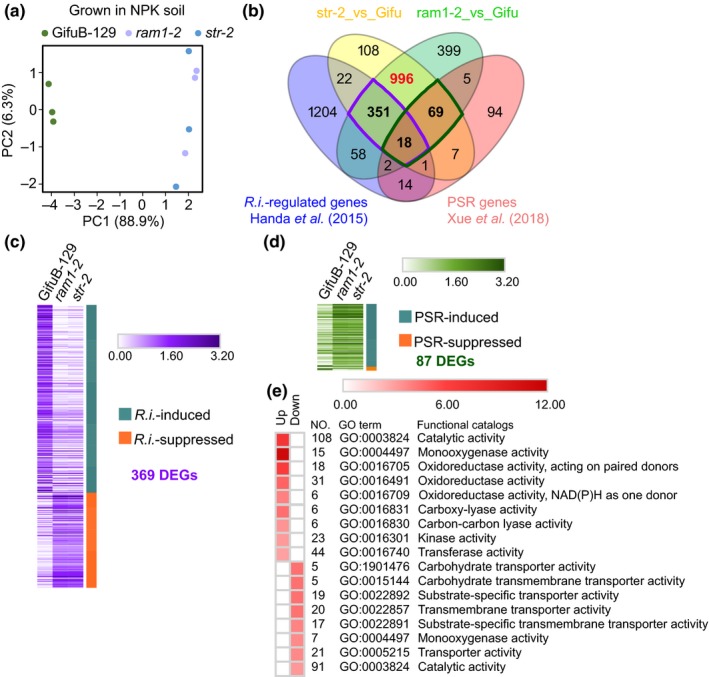
Transcriptome profiles of arbuscular mycorrhizal (AM) symbiosis defective mutants grown in NPK soil. (a) Principal components analysis of the normalized RNA‐seq counts of wild type Gifu B‐129 (WT), *ram1‐2* and *str‐2*. RNA from root samples collected from Expt 1 was used, *n* = 3. (b) Venn diagram with the overlap among differentially expressed genes (DEG) from *str‐2* or *ram1‐2* compared with WT and reference gene sets for AM symbiosis (*Rhizophagus irregularis*) and P starvation response (PSR) (log_2_ fold‐change ≥ 2 or ≤ −2, adjusted *P *≤* *0.05). (c) Heatmap of the intersect of 369 AM symbiosis‐regulated DEG. Average of log_10_ (cpm + 1) was used. (d) Heatmap of the intersect of 87 PSR‐regulated DEG. (e) Heatmap of −log_10_ *P*‐values of GO terms enriched in the remaining 996 common DEG.

To explore transcriptome profiles, roots of WT, *ram1‐2* and *str‐2* grown in NPK soil were subjected to RNA sequencing. The overall expression profile of WT was substantially different from that of *ram1‐2* and *str‐2* mutants (Fig. 2a; Tables S5 and S6). In total, 1434 DEG were common in both mutants (75.6% of DEG in ‘*ram1‐2* vs WT’ and 91.2% of DEG in ‘*str‐2* vs WT’). Of these common DEG, a total of 369 DEG (25.7%) were AM symbiosis‐regulated (Table S7), while 87 DEG (6.1%) were associated with the phosphate starvation response (PSR) (Table S8; Fig. 2b). Within the 369 AM symbiosis‐regulated common DEG, 96.7% (237/245) of AM fungi‐induced genes showed compromised transcription in mutants, while 94.4% (119/124) of normally AM fungi‐suppressed genes were expressed at a higher level (Fig. 2c), indicating that AM symbiosis was affected similarly in both mutants. Within the PSR‐regulated common DEG (87), expression of 95.1% (78/82) of PSR‐induced genes was increased in both mutants, while three out of five PSR‐suppressed genes showed reduced expression in both mutants (Fig. 2d), suggesting more pronounced PSR in arb^−^ lines. The remaining common DEG (996, i.e. 549 upregulated, 447 downregulated) were subjected to Gene Ontology enrichment analysis. The GO terms related to monooxygenase and oxidoreductase activity were significantly enriched within the upregulated gene set and transport‐related GO terms were overrepresented within the downregulated gene set (Fig. 2e), including genes encoding P, K and S transporters (Table S5), suggesting that redox processes and transport activities were disturbed in arb^−^ plants.

### Plants perturbed in AM symbiosis display altered assembly of fungal consortia

**Figure 3:**
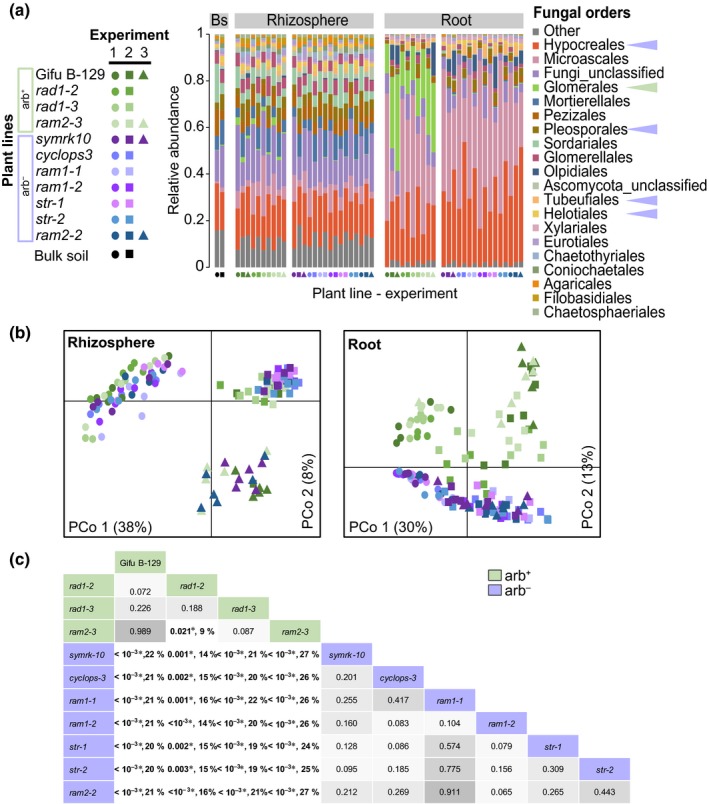
Roots of *Lotus japonicus* mycorrhizal mutants establish altered root fungal communities. (a) Relative abundance of fungal orders in bulk soil (Bs), rhizosphere and roots of *L. japonicus*. Plant lines are color‐coded with green shades for arbuscule‐forming lines (arb^+^) and blue/purple shades for lines without arbuscules (arb^−^). The three independent experiments (Experiment) are symbol‐coded. Arrowheads indicate fungal orders depleted (green) or enriched (purple) in the rhizosphere and roots of arb^−^ lines (*t*‐test, *P *<* *0.01). (b) Principal coordinates analysis on Bray‐Curtis distances. Samples are color‐ and symbol‐coded according to the legend in (a). (c) *P*‐values and percentage of variance explained by mycorrhizal status from pairwise comparisons of root fungal communities between plant lines. Bold numbers indicate significant differences between lines (PerMANOVA, *P *<* *0.05), the grey scale indicates *P*‐values < 0.05, asterisks indicate *P*‐values still significant at 0.05 after FDR correction. Results from three independent experiments are shown in (a) and (b), data from Expt 3 was excluded for the analysis in (c) because of incomplete representation of all plant lines.

We compared root‐associated fungal microbiomes of the 10 mutant lines, using high‐throughput amplicon sequencing of the fungal taxonomic marker ITS2. Fungal alpha diversity differed among compartments, with root samples being significantly less diverse than rhizosphere and soil samples, while no significant differences were observed between the plant lines (Fig. S4a). Relative abundances of fungal orders differed between arb^+^ and arb^−^ lines (Fig. 3a). The Glomerales order represented on average 22% of the fungal reads found in root samples of arb^+^ lines, while they accounted for only 0.7% of the fungal community members in roots of the arb^−^ lines (98% reduction in arb^−^ genotypes; Wilcoxon's test *P* < 10^−15^). Concomitantly with the depletion of Glomerales in arb^−^ roots, an enrichment of Ascomycetes from the orders Hypocreales (+ 50%), Pleosporales (+ 65%), Tubeufiales (+ 127%) and Helotiales (+ 138%) was observed (Wilcoxon's test *P* < 0.05). These differences occurred also but to a lesser extent in rhizospheric fungal communities (Fig. 3a). PerMANOVA analysis conducted on Bray‐Curtis distances between the samples revealed that most of the differences in fungal community structure were driven by the microhabitat type (bulk soil, rhizosphere or root endosphere) and the experiment (48% and 13% of explained variability, respectively; Table S9 and Fig. S4b). The statistically significant effect of the mycorrhizal status on fungal communities accounted for 16% of the variability in the root, 2% in the rhizosphere and 3% of the overall variability (PerMANOVA *P* < 0.05, Table S9). The arb^+^ and arb^−^ lines differed primarily in the fungal communities established within their root tissues and not around them (Table S9). This was supported by the PCoA on root fungal communities where arb^+^ and arb^−^ lines separated along the second axis capturing 13% of the variance (Fig. 3b). Plant lines with the same mycorrhizal status shared similar root fungal communities, independent of the function of the mutated gene (PerMANOVA *P* > 0.05). Pairwise comparisons of root fungal communities showed that the mycorrhizal status could explain 20–22% of variance when comparing each arb^‐^ line with WT (Fig. 3c). Within arb^−^ lines, plants having lost the capacity to establish RN symbiosis (*symrk‐10* and *cyclops‐3*) still established similar root fungal communities to the nodule‐forming arb^−^ lines, suggesting that nodulation has no effect on fungal community shifts (Fig. 3c).

To focus on the differences beyond AM fungal taxa, the statistical data analysis was also performed after removal of all Glomeromycota OTUs. Although there was an important experimental impact in root (38% of variance explained), 5% of the differences observed between root fungal communities across all 10 plant lines studied was explained by the mycorrhizal status of the plant (PerMANOVA *P* < 0.05; Table S10). This was supported in the PCoA, where root samples from arb^+^ and arb^−^ plants separated along the second axis capturing 10% of the variance (Fig. S5b), with unchanged alpha diversity (Fig. S5a). Pairwise comparisons of arb^–^ lines with WT showed similar differences, with the mycorrhizal status explaining 9–11% of variance (Fig. S5c). *rad1‐2* and *rad1‐3* exhibited a root fungal community more similar to that of *symrk‐10*,* ram1‐2* or *str‐2* after exclusion of Glomeromycota reads (Fig. S5c), suggesting an intermediate microbiome phenotype in transition from arb^+^ to arb^−^ in these lines.

### Aberrant arbuscule development correlates with depletion of AM fungi and enrichment of Ascomycota taxa in roots

**Figure 4:**
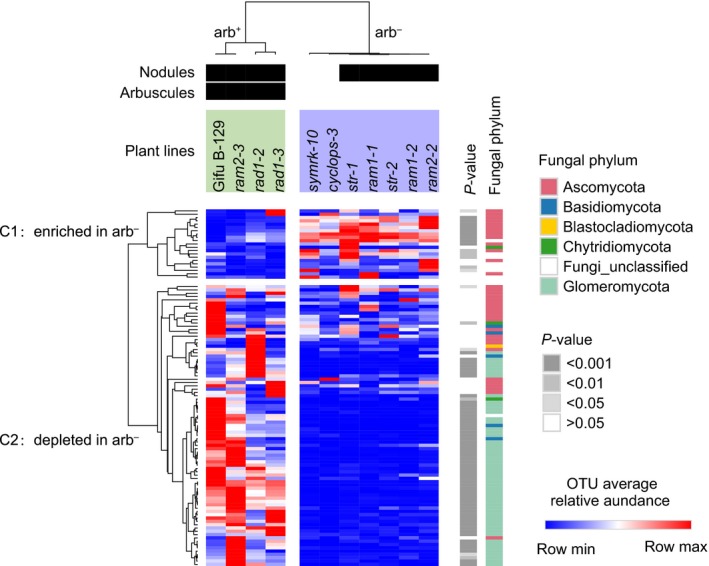
Roots of *Lotus japonicus* mycorrhizal mutants are depleted in Glomeromycota and enriched for several taxa from the Ascomycota. Heatmap showing fungal operational taxonomic units (OTUs) enriched (cluster C1) or depleted (cluster C2) in the roots of arb^−^ plant lines. OTUs were identified by differential abundance analysis (in DESeq2; *P *<* *0.05), ‘*P*‐value’ indicates results from subsequent pairwise tests comparing OTUs relative abundances in arb^+^ and arb^−^ lines (Wilcoxon's test). Arbuscules or nodule‐forming lines are indicated with black squares.

Root fungal communities were compared to identify specific fungal OTUs enriched or depleted in arb^−^ and arb^+^ plant lines. A total of 106 OTUs were identified in this analysis corresponding to 6.9% of all OTUs detected in roots (1526). Fungal OTUs depleted in the roots of arb^−^ plants (Fig. 4, cluster C2) concerned mainly taxa belonging to the Glomeromycota phylum (54). The most depleted group was OTU00005 classified as *Funneliformis* sp., representing up to 14.5% of the fungal reads in arb^+^ plants but only 0.14% in arb^−^ plants (103‐fold depletion; Wicoxon's test *P* < 10^−15^). Similar results were observed after dividing the number of OTU00005 reads by the number of plant reads in the sample (Fig. S6a). Other AM fungal genera depleted in arb^−^ root included *Septoglomus*,* Claroideoglomus*,* Glomus* and *Rhizophagus* (Table S11). Thirty‐two non‐Glomeromycota OTUs were also depleted in arb^−^ roots but to a smaller extent. A significant depletion (Wilcoxon's test *P* < 0.05 after FDR correction) was observed for only seven OTUs, which all showed low relative abundances (< 0.1%). These included two OTUs from the Chytridiomycota, one of them classified as *Spizellomyces dolichospermus* (OTU00289), two from unclassified Basidiomycota taxa (OTU00236 and OTU01541) and three Ascomycota (OTU00026 *Thelonectria olida*, OTU00063 *Emericellopsis terricola* and OTU00919 an unclassified Talaromyces).

**Figure 5:**
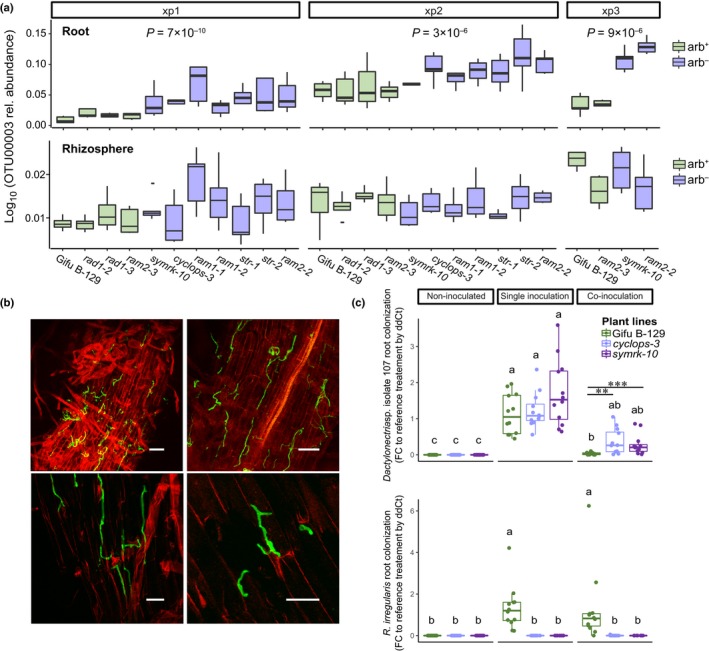
Root colonization by the arbuscular mycorrhizal (AM) fungus *Rhizophagus irregularis* inhibits colonization by a *Dactylonectria torresensis* isolate. (a) Relative abundance of *D. torresensis* OTU00003 in the root endosphere of arb^+^ and arb^−^ *Lotus japonicus* plant lines across three independent experiments. The values are inferred from fungal ITS2 sequencing data. The *P*‐value indicates the higher relative abundance in arb^−^ roots (Wilcoxon's test). In the box‐plots, horizontal lines represent the first, second (median) and third quartiles while whiskers depict the dispersion of the data (1.5 × interquartile range). (b) CLS microscopy images of wild type (WT) roots colonized by *D. torresensis*. Plant cells were stained with propidium iodide and fungal structures with WGA‐Alexa 488. Bars: 50 μm (upper panels); 20 μm (lower panels). (c) qPCR quantification of root colonization by *D. torresensis* isolate 107 and *R. irregularis* in single and co‐inoculation in a simplified sand/soil system. Data was analyzed by the 2^−ΔΔCt^ method using *L. japonicus Ubiquitin* as reference gene and ‘Single inoculation GifuB‐129’ as reference treatment. Different letters indicate significant differences between treatments and experiments (Kruskal‐Wallis followed by Dunn's test, *P *<* *0.05) and asterisks indicate significant differences between plant lines in the co‐inoculation experiment (Wilcoxon test: **, *P *<* *0.01; ***, *P *<* *0.001). Three independent experiments including four independent biological replicates were conducted (*n* = 12) with similar results (results from independent experiments are shown in Supporting information Fig. S7c,d). In the box‐plots, points represent biological replicates, horizontal lines represent the first, second (median) and third quartiles while whiskers depict the dispersion of the data (1.5 × interquartile range).

Nineteen fungal OTUs were enriched in arb^−^ roots (Fig. 4, cluster C1), corresponding to 1.2% of all OTUs detected in roots (1526). A significantly high enrichment (Wilcoxon's test *P* < 0.05 after FDR correction) was observed for nine Ascomycota taxa, including taxa with high relative abundance (> 0.1%) like OTU00015_*Titaea_maxilliformis*, OTU00080_*Cistella*_sp., OTU00087 from the Helotiales, OTU00107_*Periconia_macrospinosa* and OTU00003_*Dactylonectria_torresensis* (syn. *Ilyonectria torresensis*, the second most abundant fungal OTU in *L. japonicus* roots). Although *D. torresensis* OTU00003 was detected at similarly high levels in the rhizosphere of arb^+^ and arb^−^ plants exhibiting comparable relative abundance (3.3%; Wilcoxon's test *P* > 0.05), its relative abundance increased twofold in the root endosphere of arb^−^ plants (19.5%) relative to their arb^+^ counterparts (9.5%; Wilcoxon's test *P* < 10^−8^) (Fig. 5a; Table S11). Similar results were observed after dividing the number of OTU00003 reads by the number of plant reads in the sample (Fig. S6b). Furthermore, the fact that *D. torresensis* OTU00003 was more abundant in root relative to rhizosphere samples (Wilcoxon's test, *P* < 10^−7^) suggested a preference of this fungus for the root endosphere niche.

### *Dactylonectria torresensis* is excluded from roots harboring mature arbuscules

Screening of a collection of root‐associated fungi from *L. japonicus* growing in NPK soil yielded one *D. torresensis* isolate (isolate 107). The ‘model AM fungus’ species *R. irregularis* (with sequenced genome) which was among the taxa depleted in arb^−^ mutants (Table S11) and isolate 107 were subsequently used for studies in a gnotobiotic system. The single inoculation of isolate 107 showed that *D. torresensis* similarly colonized the roots of WT plants, *symrk‐10* and *cyclops‐3* (Figs. 5b; S7a,b), indicating that root colonization by this fungus is not directly affected by the loss of function in two common symbiosis genes. Co‐inoculation with *R. irregularis* limited root colonization by *D. torresensis* in all examined plant lines, with stronger suppression in WT relative to *cyclops‐3* and *symrk‐10* plants (Figs 5c, S7c,d) (*cyclops‐3* vs WT, Wilcoxon test *P *= 0.0011; *symrk‐10* vs WT Wilcoxon test, *P *= 0.0008). Moreover, co‐inoculation with *D. torresensis* had no impact on root colonization by the AM fungus (Wilcoxon test, *P ≥* 0.05) (Fig. 5c) or on arbuscule formation (Tukey's HSD test, *P *>* *0.05; Fig. S7a). Different results were observed with non‐*Dactylonectria* isolates 57 and 102, where co‐inoculation of the AM fungus negatively affected root colonization by both isolates independently of the arb phenotype of the plant (Fig. S8). These results suggested that both the presence of AM fungi (inoculation) and arbuscule formation trigger the exclusion of *D. torresensis* from *L. japonicus* roots.

## Discussion

Here, the impact of arbuscular mycorrhizal symbiosis establishment on root fungal communities was studied in *L. japonicus* WT and 10 mutants impaired in the CSSP genes and downstream processes. Mycorrhizal phenotypes of the investigated mutants grown in natural non‐sterile soil (Fig. 1) were similar to phenotypes observed in binary AM fungus–host interactions (Fig. S2). Unlike in binary interactions, host plants which had lost their ability to establish a functional AM symbiosis (arb^−^) exhibited reduced growth performance, reduced acquisition of P and other elements, and lower biomass in natural soil (Fig. S3). Comparative transcriptomic analysis further showed the repression of phosphate, potassium and sulfate transporter genes corresponding with a nutrient imbalance (P, K, S) in the mutants, as well as the induction of PSR genes accompanied by changes in redox homeostasis (Fig. 2; Tables S5 and S6). These results allude to the impact of arbuscule functioning on the transcriptional control of nutrient uptake, redox status and plant growth in natural soil conditions.

Growing in natural soil, arb^+^ and arb^−^ lines harbored distinguishable fungal communities differing mainly in Glomeromycota taxa. Within a broad spectrum of Glomeromycota taxa (Fig. 3), arb^+^ lines showed some preference for specific taxa, like the AM fungus *Funneliformis* sp. (OTU00005; Table S11). When non‐AM fungal communities in arb^+^ roots were compared with arb^−^ plants, we observed that all arb^−^ plants exhibited similarly altered root endophytic fungal microbiota (Fig. S5; Table S11). Although these differences were robust, i.e. they were stable across different arb^+^ and arb^−^ plant lines and across independent experiments, they were relatively small, with the arb phenotype explaining 5% of the observed variance in roots (Table S10). In comparison to other microbiome studies, this ‘effect size’ remained in the lower range of what has been observed for differences between plant ecotypes (5–12% of explained variance in root bacterial community) (Dombrowski *et al*., 2017) or for the disruption of genes essential for the nitrogen‐fixing symbiosis in *L. japonicus* (9.8% of explained variance in root bacterial community) (Zgadzaj *et al*., 2016). Nonetheless, when these results are put into perspective, considering that ‘microhabitat’ accounted for 50% of the variability and ‘experiment’ for 13% (Table S10), one must concede that the mycorrhizal status is of comparatively limited importance with respect to non‐AM fungal communities. It was reported that silencing of the CSSP gene *CCaMK* in field‐grown *Nicotiana attenuata* does not significantly affect the root‐associated bacterial and fungal communities (Groten *et al*., 2015). This could be explained by the fact that the detection of small effects needs repeated experiments including large enough numbers of replicates and plant lines to correct for confounding effects and assure adequate statistical power in the analysis, which was rather limited in the previous study.

Our results showed that 6.9% of all the OTUs detected in roots were affected by AM symbiosis disruption. As expected, most of the OTUs depleted in arb^−^ roots belonged to the Glomeromycota phylum encompassing all AM fungi. Interestingly, 32 non‐AM fungal taxa were also depleted, suggesting that AM symbiosis disruption limited root colonization by these fungi through an as yet unknown mechanism (Fig. 4; Table S11). It has been hypothesized that non‐symbiotic microorganisms can highjack the AM symbiosis pathway to effectively colonize plant tissues, but experimental outcomes were of a mixed nature (Wang *et al*., 2012; Huisman *et al*., 2015; Rey *et al*., 2015). Our observation that few non‐Glomeromycota fungal taxa were consistently depleted in the roots of arb^−^ plants is in line with this hypothesis (Fig. 4), suggesting that microbial hijacking occurred, with some root endophytic fungi naturally occurring in the studied soil. However, we cannot exclude the possibility that these taxa are depleted because of a functional interaction with AM fungi. For example, among the fungal taxa depleted in the roots of arb^−^ plants, the presence of a *Spizellomyces* taxon is noteworthy. This chytridiomycete has been shown to live in association with AM fungal spores, and its lower abundance in arb^−^ roots may be explained by a trophic link between this fungus (as parasite or saprophyte) and the AM fungus (Paulitz & Menge, 1984).

The consistent enrichment of 19 Ascomycota taxa from the Hypocreales, Tubeufilales, Helotiales, and Pleosporales was observed in arb^−^ samples, suggesting that a non‐functional AM symbiosis enhanced root colonization by these fungi. Helotiales fungi have been shown to accumulate in roots of non‐mycorrhizal hosts deprived of phosphate and to transport phosphate from the root environment to their host plant (Almario *et al*., 2017; Fabianska *et al*., 2019). A functional relationship between the mycorrhizal status and nonmycorrhizal fungi with implications in nutrient acquisition awaits consideration in future work. We hypothesize that AM symbiosis impairment can facilitate colonization of the roots by distinct non‐AM fungi via two non‐exclusive mechanisms associated with: (i) direct AM fungus–microbe interactions, which could include growth inhibition by antagonistic interactions through the release of antimicrobial substances (Filion *et al*., 1999) or through competition for resources (Green *et al*., 1999); or (ii) plant‐mediated AM fungal effects associated with the activation and/or priming of plant defense (Gerlach *et al*., 2015). Plant‐protecting activities of AM fungi against fungal pathogens have been described (Azcón‐Aguilar & Barea, 1997), which suggests that AM fungi trigger changes affecting other microbes. Although none of the enriched taxa identified in our study are known to be pathogenic on *L. japonicus*, we could imagine that conserved mechanisms including immune responses underlying interactions of *L. japonicus* with pathogens and commensals could explain these observations (García‐Garrido & Ocampo, 2002; Lebeis *et al*., 2015). Alternatively, observed robust shifts in root‐associated fungal communities could be the consequence of a mounted PSR in the plant (Castrillo *et al*., 2017; Fabianska *et al*., 2019) and derived metabolic consequences including, e.g. redox homeostasis (Fig. 2).

The OTU00003 classified as *D. torresensis* was the second most abundant root OTU in arb^+^ plants and was two‐times more abundant in the roots of arb^−^ lines (Fig. 5a; Table S11). *D. torresensis* belongs to the Cylindrocarpon complex in the Hypocreales and has been associated with black foot disease in grapevines, strawberries and other plants (Lombard *et al*., 2014), but in our experiments isolate *D. torresensis* 107 was non‐symptomatic on *L. japonicus* at the stage of harvest (Fig. S7b). Root colonization by *D. torresensis* 107 was consistently suppressed upon co‐inoculation with the AM fungus *R. irregularis* and this effect was clearly stronger on mycorrhized arb^+^ plants where *D. torresensis* 107 DNA was almost undetectable (Fig. 5c). By contrast, complete suppression of root colonization by non‐*Dactylonectria* isolates 57 and 102 through AM fungal hyphae occurred independent of the mycorrhizal phenotype of the roots (Fig. S8). This may be explained by microbe–microbe interactions and antimicrobial substances released by germinated AM fungal spores, or extraradical and/or intraradical hyphae (Filion *et al*., 1999). Moreover, we observed substantial variation in fungal susceptibility to these suppressive effects of the AM fungus (Figs 5c; S8). Consistent with our findings, a recent study on the root‐associated fungal microbiota of Australian palm trees showed that reduced AM fungal colonization of roots was correlated with higher abundance of *Dactylonectria* taxa (Osborne *et al*., 2018). Together, these studies suggest that this antagonistic interaction between AM fungi and *Dactylonectria* is conserved across plant species and geographical locations.

Our results on mycorrhizal root microbiome structure and host physiology in *L. japonicus* AM symbiosis mutants suggest a role of symbiosis functionality in structuring root microbiota. Dysfunctional AM symbiosis caused large physiological and transcriptional changes in the mycorrhizal (arb^−^) host plants, including strongly reduced mycorrhizal P uptake and host plant biomass, accompanied by enhanced expression of PSR and redox‐related genes and reduced expression of genes involved in nutrient transport. AM symbiosis dysfunctionality strongly reduced abundance of AM fungi and had a limited effect on non‐AM fungal taxa. Moreover, our results suggested that the observed fungal colonization dynamics of *L. japonicus* were driven by the formation and functionality of symbiosomes (Gutjahr & Parniske, 2017). We propose a model in which, as a first line of root microbiome establishment, the CSSP facilitates cellular reprogramming of the root by enabling preferential colonization by AM fungi, which form a dense network of intraradical hyphae with arbuscules (arb^+^ phenotype), accompanied by a set of non‐AM fungi. The controlled accommodation of fungal symbionts displaces other fungal taxa (e.g. Ascomycetes) likely through niche competition. The robustness of the mycorrhizal microbiome is maintained through transcriptional regulation of reciprocal exchange of carbon and P at the AM symbiosis interface (Bravo *et al*., 2017; Jiang *et al*., 2017, 2018; Keymer *et al*., 2017; Luginbuehl *et al*., 2017; Xue *et al*., 2018), which reduces P starvation stress and associated metabolic and developmental processes in the host, working as a second line for microbial accommodation. Future work will aim at further elucidating host plant and mycorrhizal microbiota interdependencies, which should provide tools for the manipulation of the root microbiome for beneficial outcomes.

## Author contributions

LX, JA and MB planned and designed the research. LX, JA and IF performed the experiments. LX, JA, IF and GS carried out the data analysis. LX, JA and MB wrote the manuscript. LX and JA contributed equally to this work.

## Supporting information

Please note: Wiley Blackwell are not responsible for the content or functionality of any Supporting Information supplied by the authors. Any queries (other than missing material) should be directed to the *New Phytologist* Central Office.

**Fig. S1** Positions of mutation.
**Fig. S2** Mycorrhizal phenotype of the plant lines in low Pi sand/soil gnotobiotic system upon *R. irregularis* inoculation.
**Fig. S3** Shoot fresh biomass (a) and shoot element concentration quantified by ICP‐MS (b) of the different lines grown in the natural soil system (Expt 1).
**Fig. S4** Fungal community alpha diversity and structure in the different plant lines.
**Fig. S5** Fungal community alpha diversity and structure in the different plant lines after the exclusion of Glomeromycota OTUs.
**Fig. S6** Abundance of OTU00005 *Funneliformis* sp. (a) and *D. torresensis* OTU00003 (b) in the roots of arb^+^ and arb^−^ *L. japonicus*.
**Fig. S7** Quantification of root colonization and fresh shoot weight co‐inoculated with *R. irregularis* and *D. torresensis* 107.
**Fig. S8** Presence of *R. irregularis* inhibits colonization by non‐*Dactylonectria* fungal isolates.
**Methods S1** Supplementary methods
**Table S1** Plant lines used in this study.
**Table S2** Soil characteristics.
**Table S3** Experimental design.
**Table S4** Primers used in this study.
**Table S5** DEG in roots of *ram1‐2* mutant vs WT grown in NPK soil.
**Table S6** DEG in roots of *str‐2* mutant vs WT grown in NPK soil.
**Table S7** DEG in inoculated vs non‐inoculated roots of WT with *R. irregularis* (Handa *et al*., 2015).
**Table S8** DEG in roots of WT grown in sand/soil mixture and fertilized with low Pi (5 μM) vs high Pi (7.5 mM) (Xue *et al*., 2018).
**Table S9** Differences of alpha diversity (ANOVA on Shannon's H index) and structure (PerMANOVA on Bray‐Curtis dissimilarities) of fungal communities in the different plant lines and compartments.
**Table S10** Differences of alpha diversity (ANOVA on Shannon's H index) and structure (PerMANOVA on Bray‐Curtis dissimilarities) of fungal communities in the different plant lines and compartments after exclusion of Glomeromycota OTUs.
**Table S11** Fungal OTUs enriched and depleted in the roots of arb^−^ *L. japonicus* lines (see Fig. 4).
