# Supplementary material for: Dysfunction in the arbuscular mycorrhizal symbiosis has consistent but small effects on the establishment of the fungal microbiota in Lotus japonicus
Source: New Phytol. 2019 Jul 2;224(1):409–20. doi: 10.1111/nph.15958 (PMC6773208; doi:10.1111/nph.15958)
Supplement: Supplementary file 1 — Fig. S1 Positions of mutation. Fig. S2 Mycorrhizal phenotype of the plant lines in low Pi sand/soil gnotobiotic system upon R. irregularis inoculation. Fig. S3 Shoot fresh biomass (a) and shoot element concentration quantified by ICP‐MS (b) of the different lines grown in the natural soil system (Expt 1). Fig. S4 Fungal community alpha diversity and structure in the different plant lines. Fig. S5 Fungal community alpha diversity and structure in the different plant lines after the exclusion of Glomeromycota OTUs. Fig. S6 Abundance of OTU00005 Funneliformis sp. (a) and D. torresensis OTU00003 (b) in the roots of arb+ and arb− L. japonicus. Fig. S7 Quantification of root colonization and fresh shoot weight co‐inoculated with R. irregularis and D. torresensis 107. Fig. S8 Presence of R. irregularis inhibits colonization by non‐Dactylonectria fungal isolates. Methods S1 Supplementary methods Table S1 Plant lines used in this study. Table S2 Soil characteristics. Table S3 Experimental design. Table S4 Primers used in this study. Table S5 DEG in roots of ram1‐2 mutant vs WT grown in NPK soil. Table S6 DEG in roots of str‐2 mutant vs WT grown in NPK soil. Table S7 DEG in inoculated vs non‐inoculated roots of WT with R. irregularis (Handa et al., 2015). Table S8 DEG in roots of WT grown in sand/soil mixture and fertilized with low Pi (5 μM) vs high Pi (7.5 mM) (Xue et al., 2018). Table S9 Differences of alpha diversity (ANOVA on Shannon's H index) and structure (PerMANOVA on Bray‐Curtis dissimilarities) of fungal communities in the different plant lines and compartments. Table S10 Differences of alpha diversity (ANOVA on Shannon's H index) and structure (PerMANOVA on Bray‐Curtis dissimilarities) of fungal communities in the different plant lines and compartments after exclusion of Glomeromycota OTUs. Table S11 Fungal OTUs enriched and depleted in the roots of arb− L. japonicus lines (see Fig. 4). [file NPH-224-409-s001.zip › nph15958-sup-0001-Supinfo.pdf]

Supporting Information

Article title: **Dysfunction in the arbuscular mycorrhizal symbiosis has consistent but small effects on the establishment of the fungal microbiota *in Lotus japonicus***

Authors: Li Xue, Juliana Almario, Izabela Fabiańska, Georgios Saridis and Marcel Bucher

Accepted: 15 May 2019

Methods S1

Root and rhizosphere sampling for ITS2 sequencing

Rhizosphere and root samples were collected using a fractionation method described previously (Almario *et al.*, 2017), involving the recovery of the rhizosphere soil after a first wash of the root system and a thorough cleaning of the root samples through successive washes and sonication to collect root endosphere samples. Bulk soil (Bs) was harvested from the center of unplanted pots, about 3 cm underneath the soil surface. DNA was extracted from the samples using the FastDNA SPIN Kit for Soil (MP Biomedicals, Solon, USA) according to the manufacturer's instructions. DNA samples were diluted to 4 ng/µL DNA and used for ITS2 library preparation. Libraries were prepared using ITS2 primers ITS9/ITS4 and were sequenced at the Cologne Center for Genomics (CCG) on a MiSeq instrument using the Illumina V3 kit (Illumina, CA, USA) producing 2 X 300 paired-end reads, with 3% PhiX control.

Recovery of root fungal isolates from plants grown in NPK soil

Roots of six-weeks-old L. japonicus plants growing on NPK soil were washed with sterile water and 1cm-long root pieces were placed onto MYP agar plates. Plates were incubated at 22 °C in the dark for two weeks and growing fungi were sub-cultured on new MYP agar plates. Fungal DNA was extracted and was used for ITS amplification and sequencing using primers ITS1/ITS4 as described (Almario *et al*., 2017). A fungal isolate (number 107) matching the described *Dactylonectria torresensis* OTU00003 was identified and used in further inoculation experiments.

RNA extraction and qRT-PCR

RNA from roots was extracted using the NucleoSpin RNA Plant extraction kit (Macherey Nagel, Dueren, Germany) according to the manufacturer's instructions. Genomic DNA was digested with DNase (Promega, USA) according to the manufacturer's instructions and DNA-free RNA samples were reverse transcribed into cDNA using the Thermo Scientific RevertAid H Minus Reverse Transcriptase (Applied Biosystems, Cheshire, UK). Transcript levels were determined by quantitative real-time PCR in Quantstudio 5 (Thermo Fisher) using SYBR green PCR master mix (Applied Biosystems, Cheshire, UK). Relative gene expression levels were calculated by the $2^{-\Delta Ct}$ method, using *L. japonicus ubiquitin* expression as reference. All primers used are listed in Table S4.

ICP-MS analysis for shoot multi-elemental composition

Inductively coupled plasma mass spectrometry (ICP-MS) was used to determine the concentration of P and other elements in the plant shoot (Gerlach *et al.*, 2015). Briefly, 0.1 g of dried shoot material was digested in a mixture of 4 mL concentrated HNO_3 (67%) and 2 mL H_2O_2 (30%) in a microwave (Anton Paar, Multiwave 3000). The microwave program started with a power ramp of 10 min followed by 30 min at 1400 W, finishing with 15 min of cooling. After dilution with ddH_2O to 30 mL, shoot P concentration was detected by an Agilent 7700 ICP-MS instrument (Agilent Technologies, Tokyo, Japan) following the manufacturer's instructions.

Staining of fungal structures in plant roots and scoring of AM fungal colonization

In order to quantify root AM fungal colonization and overall fungal colonization of roots, fungal structures in plant roots were stained in PBS buffer with 1μg/mL WGA-Alexa Fluor 488 conjugate, as described previously (Xue *et al.*, 2015).

Gene ID

Genes used in this article can be found in the V3.0 *L. japonicus* database (http://www.kazusa.or.jp/lotus/) or Genbank (https://www.ncbi.nlm.nih.gov/nuccore/): *SYMRK* (AP004579.1/AF492655.1/Lj2g3v1467920.1), *CYCLOPS* (AP009158.1/EF569221.1,

Lj2g3v1549600.1), *RAD1* (KU563537.1/ Lj4g3v1389110.1), *STR* (AB830531/ Lj4g3v3115140.1), *RAM2* (KX823334.1), *RAM1* (KU500627.1/ KU557503.1).

Fig. S1 Positions of mutation. Gene structures and positions of insertions or mutations in *RAM1*, *RAM2*, *RAD1*, *SYMRK*, *CYCLOPS* and *STR* in the different plant lines. Black arrows indicate the 5' to 3' direction of the LORE1a transposon insertion. Gray bars represent the coding sequence and back lines represent the introns.

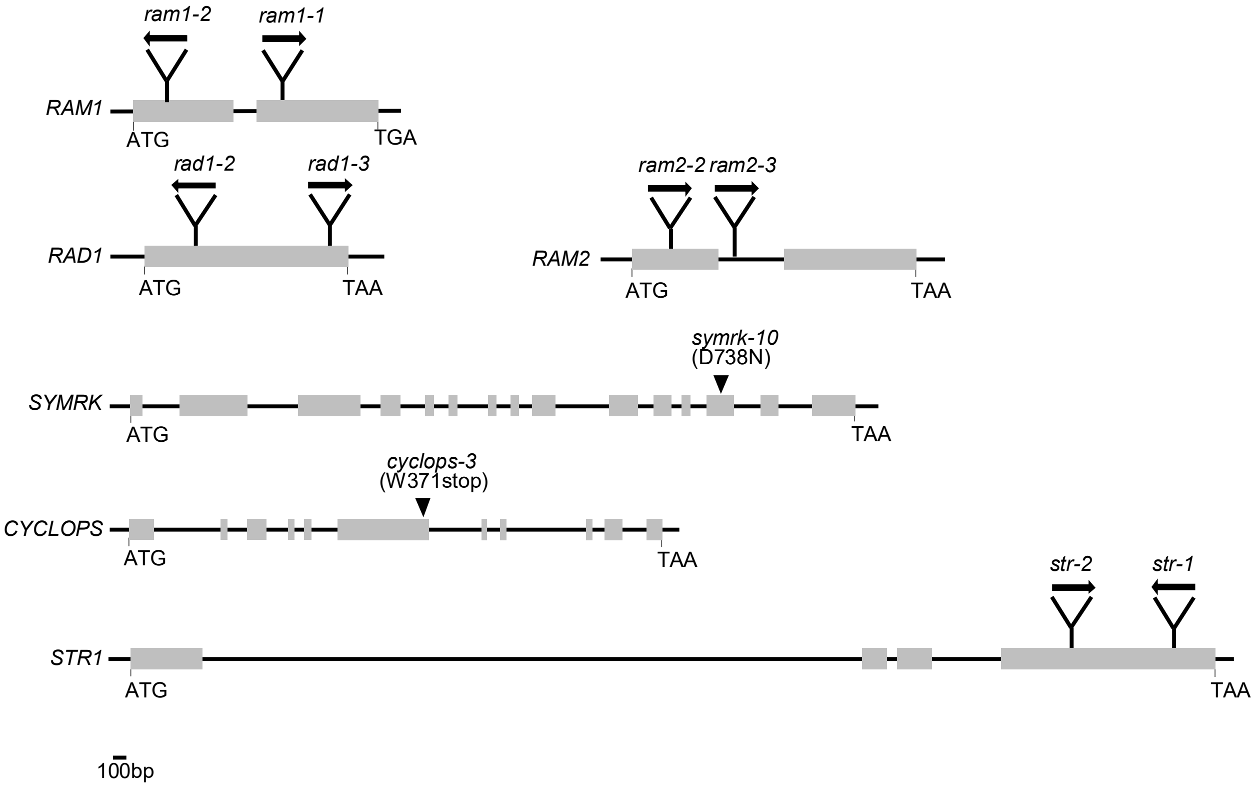

Fig. S2 Mycorrhizal phenotype of the plant lines in low Pi sand/soil gnotobiotic system upon *R. irregularis* inoculation. (a) CLSM images of root colonization by the AM fungus *R. irregularis*. The bar indicates 50μm. Well-developed arbuscules are marked with red arrows. (b) Percentage of plant roots comprising only fungal hyphae (H); hyphae and arbuscules (A+H); only vesicles (V); hyphae, vesicles and arbuscules (A+V+H); or any fungal structure (Colonization). No arbuscules or aberrant arbuscules were observed for lines noted ‘arb^{-}’. Different letters indicate significant differences between the plant lines within each category (ANOVA followed by Tukey’s HSD test, $P < 0.05$, n=3-4) (c) Expression of the plant AM symbiosis marker gene *LjPT4*. ANOVA followed by Tukey’s HSD test was performed within categories of lowP and lowP+*R. irregularis*, respectively. (d) Shoot P concentration measured by ICP-MS. (e) Plant shoot fresh weight. In panels (c) to (e), different letters indicate significant differences between the plant lines (ANOVA followed by Tukey’s HSD test, $P < 0.05$, n=3-4).

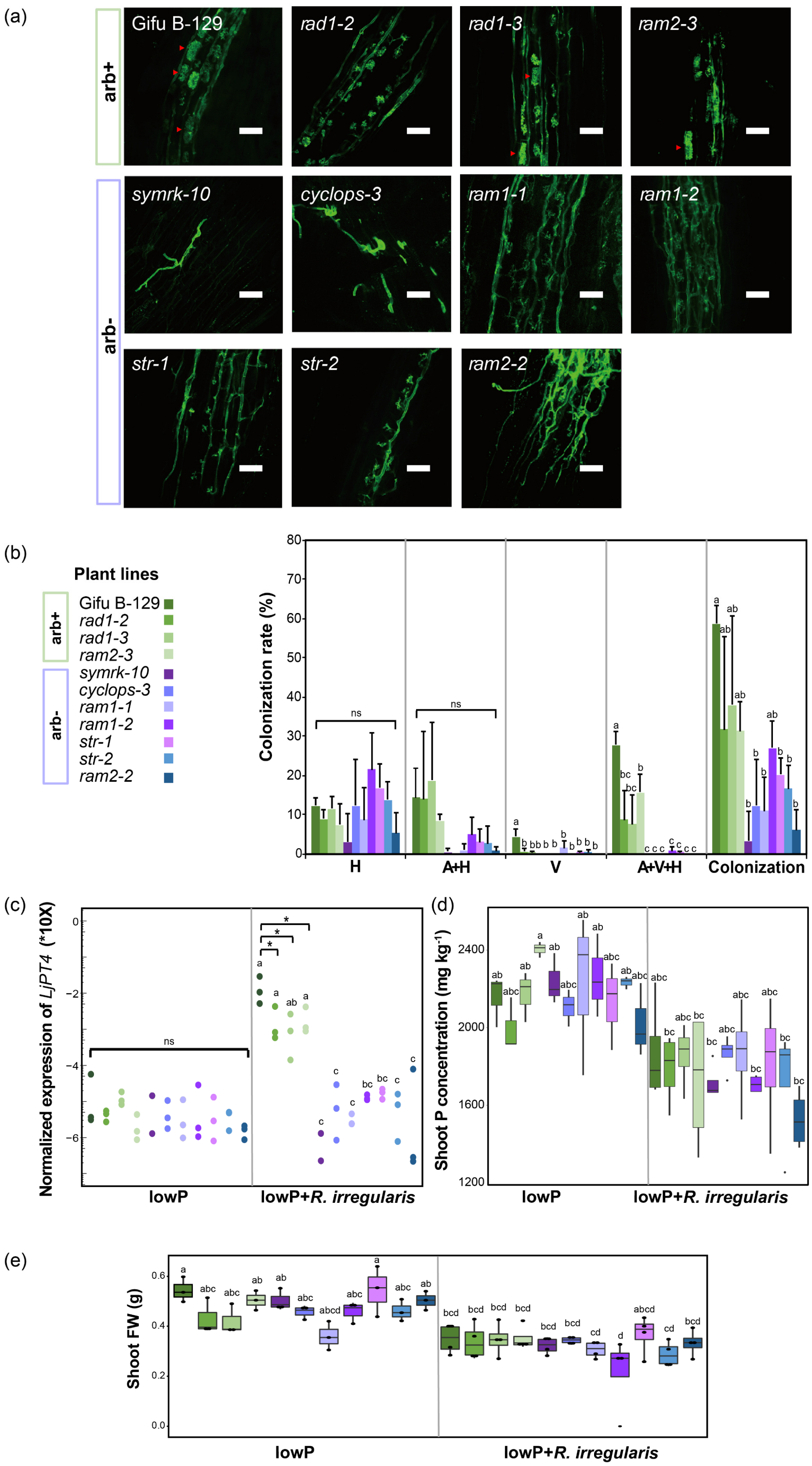
(a)
Gifu B-129
rad1-2
rad1-3
ram2-3
arb+
symrk-10
cyclops-3
ram1-1
ram1-2
arb-
str-1
str-2
ram2-2
(b)
Plant lines
arb+
Gifu B-129
rad1-2
rad1-3
ram2-3
arb-
symrk-10
cyclops-3
ram1-1
ram1-2
str-1
str-2
ram2-2
Colonization rate (%)
H
A+H
V
A+V+H
Colonization
ns
(c)
Normalized expression of LjPT4 (*10X)
lowP
lowP+R. irregularis
ns
(d)
Shoot P concentration (mg kg-1)
lowP
lowP+R. irregularis
(e)
Shoot FW (g)
lowP
lowP+R. irregularis

Fig. S3 Shoot fresh biomass (a) and shoot elemental concentration quantified by ICP-MS (b) of the different lines grown in the natural soil system (Exp 1). Different letters indicate significant differences between the plant lines (ANOVA followed by Tukey's HSD test, $P < 0.05$, n=5-6).

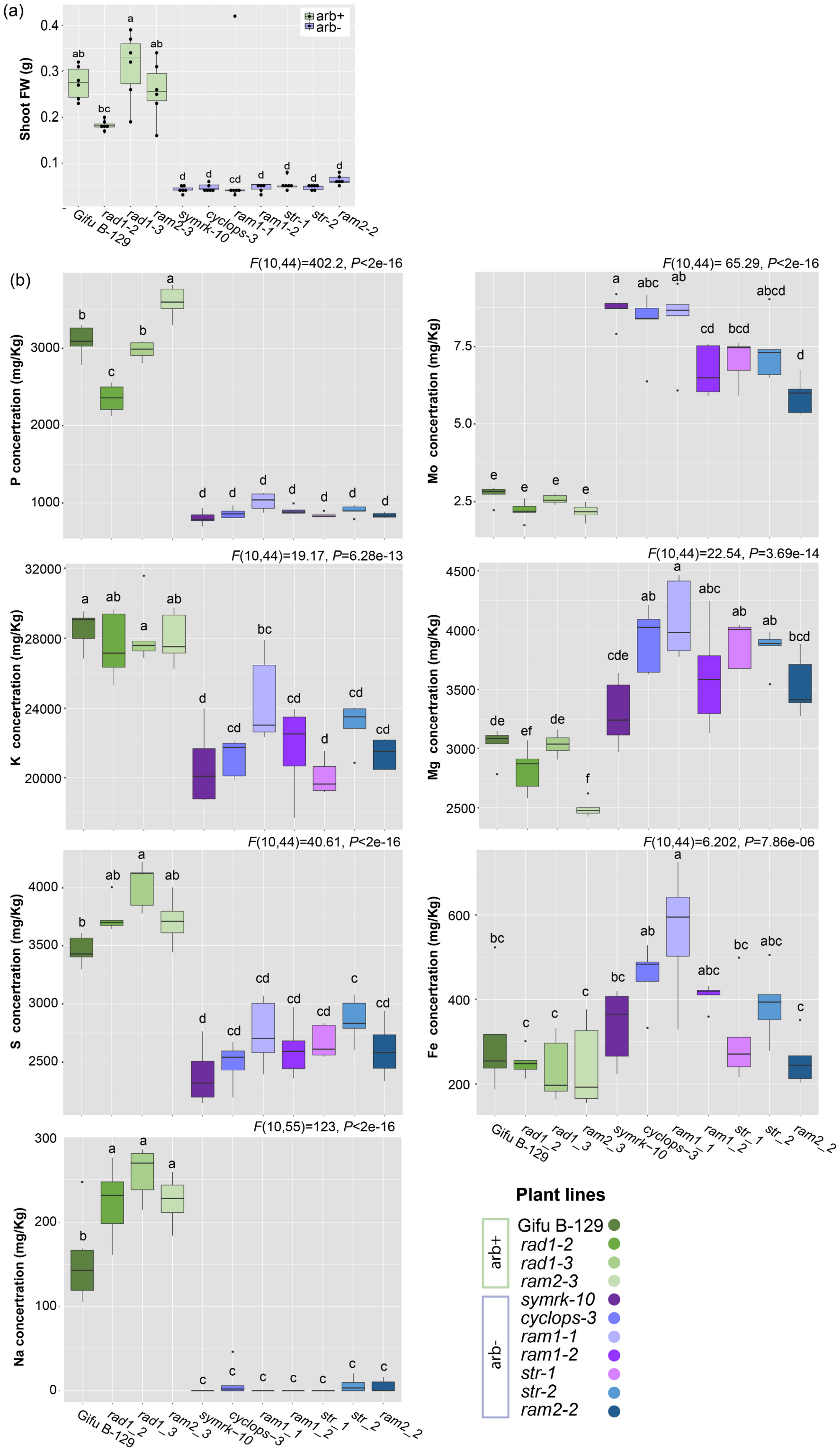

Fig. S4 Fungal community alpha diversity and structure in the different plant lines. (a) Fungal alpha diversity estimated by Shannon's index. (b) PCoA on Bray-Curtis distances between fungal communities. The results from three independent experiments are shown. Glomeromycota OTUs were included.

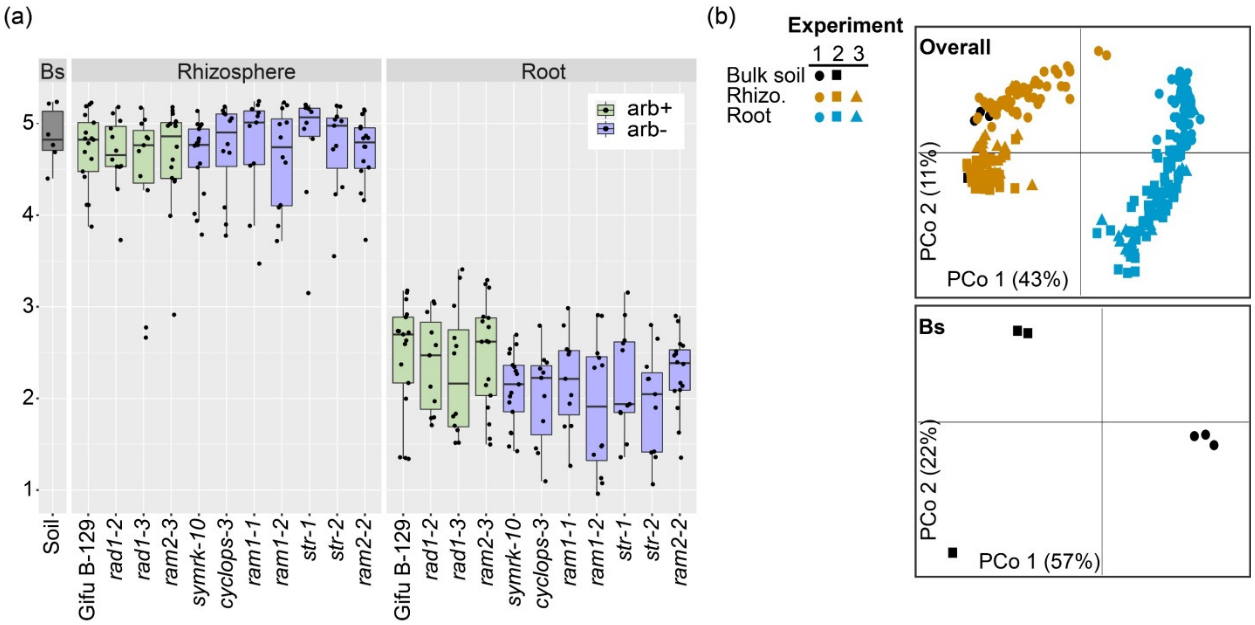

Fig. S5 Fungal community alpha diversity and structure in the different plant lines after the exclusion of Glomeromycota OTUs. (a) Fungal alpha diversity estimated by Shannon's index. (b) PCoA on Bray-Curtis distances between fungal communities. The results from three independent experiments are shown. (c) *P*-values and the variance explained by mycorrhizal status from pairwise comparisons of root fungal communities between plant lines. Bold numbers indicate significant difference between lines and the percentage of the difference explained by mycorrhizal status (PerMANOVA, $P < 0.05$). The grey scale is related to the *P*-values lower than 0.05, asterisks indicate *P*-values still significant at 0.05 after FDR correction. Results from three independent experiments are shown in (a) and (b), data from experiment 3 was excluded for the analysis in panel (c) because of the incomplete experimental set-up (n=5-6 for each plant line in each experiment).

(a)

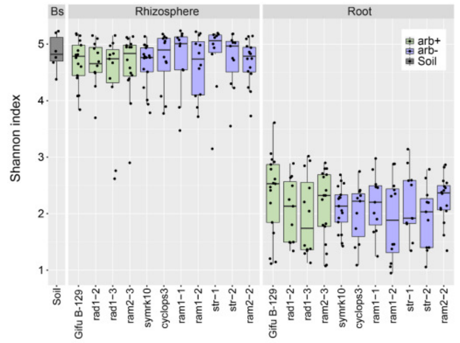

(b)

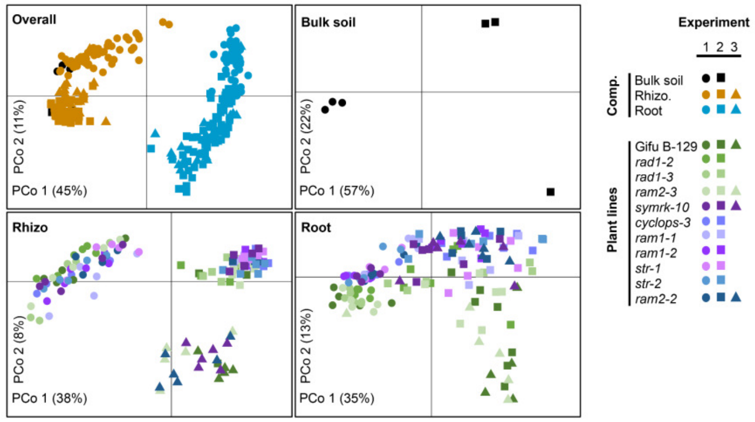

(c)

	Gifu B-129	*rad1-2*	*rad1-3*	*ram2-3*	*symrk-10*	*cyclops-3*	*ram1-1*	*ram1-2*	*str-1*	*str-2*
rad1-2	0.200									
rad1-3	0.308	0.313								
ram2-3	0.992	0.185	0.087							
symrk-10	**0.009*, 10 %**	0.089	**0.033, 7 %**	**0.007*, 12 %**						
cyclops-3	**0.006*, 11 %**	**0.017, 10 %**	**0.016, 10 %**	**0.005*, 13 %**	0.199					
ram1-1	**0.008*, 10 %**	**0.024, 10 %**	**0.018, 10 %**	**0.006*, 12 %**	0.267	0.410				
ram1-2	**0.007*, 10 %**	**0.045, 7 %**	**0.033, 7 %**	**0.004*, 11 %**	0.161	0.080	0.105			
str-1	**0.013, 9 %**	**0.023, 9 %**	**0.030, 8 %**	**0.009*, 10 %**	0.135	0.086	0.571	0.075		
str-2	**0.015, 10 %**	**0.034, 9 %**	**0.041, 8 %**	**0.011*, 11 %**	0.100	0.188	0.779	0.155	0.295	
ram2-2	**0.008*, 10 %**	**0.030, 9 %**	**0.037, 9 %**	**0.005*, 12 %**	0.218	0.282	0.916	0.065	0.277	0.457

arb+
arb-

Fig. S6 Abundance of OTU00005 *Funneliformis* sp. (a) and *D. torresensis* OTU00003 (b) in the roots of arb$^+$ and arb$^-$ *L. japonicus*. The abundance of each OTU is expressed as a ratio between the number of fungal OTU ITS2 reads divided by the number of plant ITS2 reads per sample. (a) *P*-values indicate a lower abundance of OTU00005 in arb$^-$ roots (Wilcoxon's test). (b) *P*-values indicate a higher abundance of OTU00003 in arb$^-$ roots (Wilcoxon's test). Values are inferred from fungal ITS2 sequencing data from three independent experiments (n=5-6 for each plant line in each experiment).

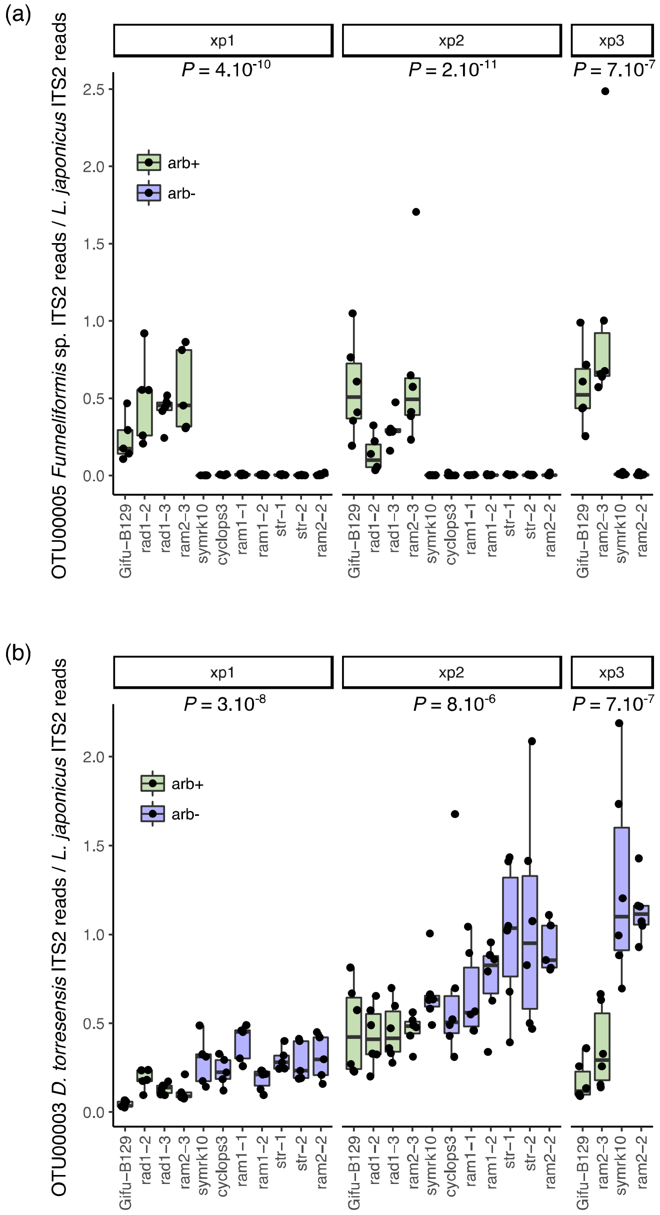

Fig. S7 Quantification of root colonization and fresh shoot weight co-inoculated with *R. irregularis* and *D. torresensis* 107. (a) Fungal colonization was analyzed in one of the three inoculation experiments (Experiment 2. ANOVA followed by Tukey's HSD test, n=4, $P<0.05$). (b) Fresh shoot weight (ANOVA followed by Tukey's HSD test, n=16, $P<0.05$). Different letters indicate significant differences between the treatments. Three experiments were performed independently with similar results. (c and d) qPCR quantification of root colonization by *D. torresensis* isolate 107 and *R. irregularis* in single- and co-inoculation in a simplified sand-soil system (three independent experiments; four biological replicates per experiment). (c) Data shown are fold changes (FC) calculated by the $2^{-\Delta Ct}$ method using the *L. japonicus Ubiquitin* gene as reference. Different letters indicate significant differences between treatments within each experiment (ANOVA followed by Tukey's HSD) and asterisks indicate significant differences between plant lines in the co-inoculation experiment (Wilcoxon test, $P<0.05$). (d) Data shown are FC calculated by the $2^{-\Delta\Delta Ct}$ method using *L. japonicus Ubiquitin* as reference gene and 'Single inoculation Gifu B-129' as reference treatment. Different letters indicate significant differences between treatments and experiments (Kruskal-Wallis followed by Dunn's test) and asterisks indicate significant differences between plant lines in the co-inoculation experiment (Wilcoxon test, $P<0.05$). Results from three independent experiments including four biological replicates are shown (n=4).

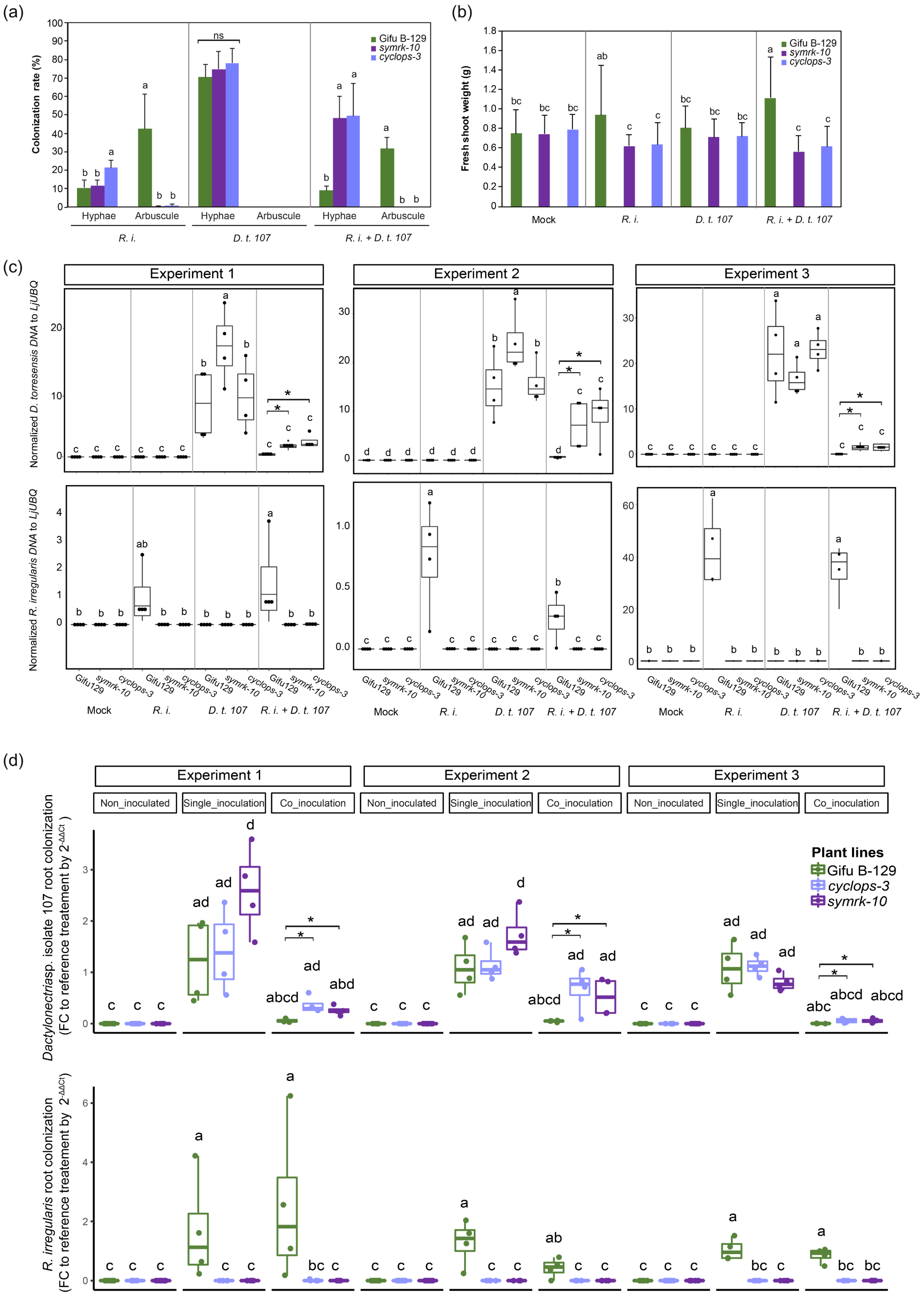
(a)
Colonization rate (%)
Gifu B-129
symrk-10
cyclops-3
ns
Hyphae
Arbuscule
R. i.
D. t. 107
R. i. + D. t. 107
(b)
Fresh shoot weight (g)
Mock
(c)
Experiment 1
Experiment 2
Experiment 3
Normalized D. torresensis DNA to LjUBQ
Normalized R. irregularis DNA to LjUBQ
Gifu129
symrk-10
cyclops-3
(d)
Non_inoculated
Single_inoculation
Co_inoculation
Dactylonectriasp. isolate 107 root colonization
(FC to reference treatement by 2^-ΔΔCt)
R. irregularis root colonization
(FC to reference treatement by 2^-ΔΔCt)
Plant lines

Fig. S8 Presence of *R. irregularis* inhibits colonization by non-*Dactylonectria* fungal isolates. qPCR quantification of root colonization by isolates I57, I102 and *R. irregularis* in single and co-inoculation in a sand-soil gnotobiotic system. Data was analyzed by the $2^{-\Delta\Delta Ct}$ method using *L. japonicus Ubiquitin* as reference gene and 'Single inoculation Gifu B-129' as reference treatment. Different letters indicate significant differences between treatments and experiments (Kruskal-Wallis followed by Dunn's test, $P < 0.05$). The experiment was conducted once with each fungal isolate (n=4). The experimental set-up is described in Table S3.

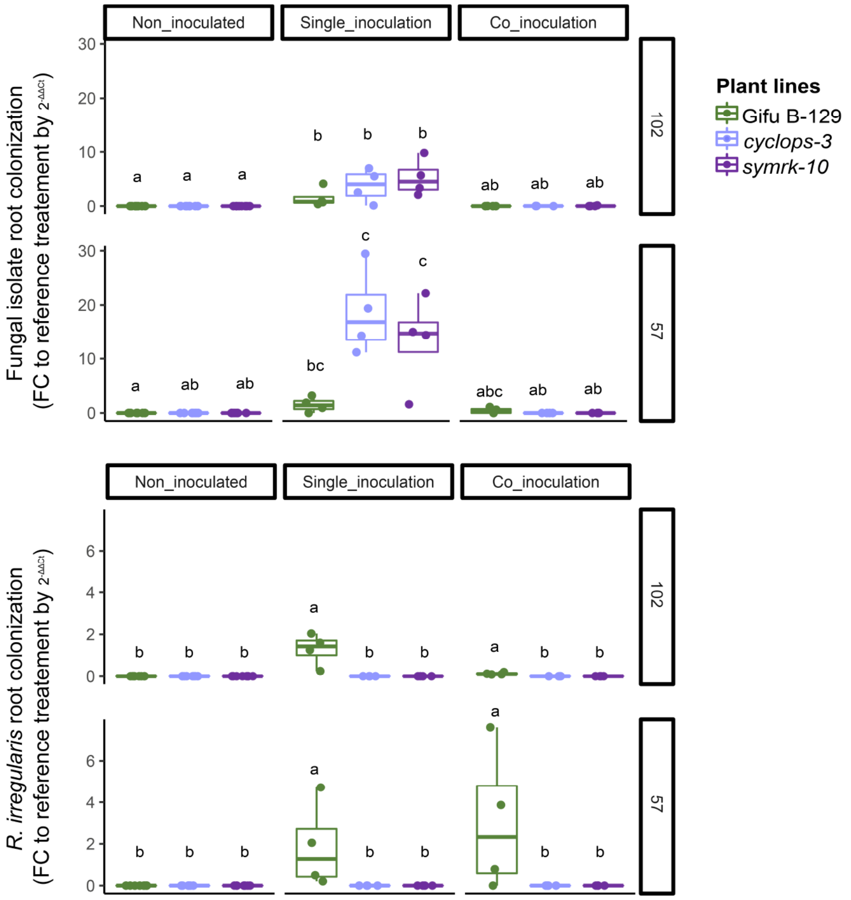

Table S1 Plant lines used in this study. All mutant lines were in Gifu B-129 background.

Gene Name	Encoding protein	Mutant lines	Nodule formation	Arbuscule formation	References of mutants
SYMRK	Receptor-like kinase	*symrk-10*	-	-	(Yoshida & Parniske, 2005)
CYCLOPS	Coiled-coil domain containing protein	*cyclops-3*	-	-	(Yano *et al.*, 2008)
RAM1	GRAS protein (Gobbato *et al.*, 2012) (Rich *et al.*, 2015) (Park *et al.*, 2015)	*ram1-1*	+	-	(Xue *et al.*, 2015)
		ram1-2	+	-	
RAD1	GRAS protein (Xue *et al.*, 2015) (Rey *et al.*, 2017)	*rad1-2*	+	+	(Xue *et al.*, 2015)
		rad1-3	+	+	
RAM2	Glycerol-3-phosphate acyl transferase (Wang *et al.*, 2012) (Keymer *et al.*, 2017)	*ram2-2*	+	-	(Keymer *et al.*, 2017)
		ram2-3	+	+	In this work
STR	Half-ABC transporter (Zhang *et al.*, 2010) (Gutjahr *et al.*, 2012)	*str-1*	+	-	In this work
		str-2	+	-	In this work

Table S2 Soil characteristics. The NPK soil was collected in 2013 from a field of the research station Agroscope in Zurich. The analysis was conducted by the lbu (Laboratory for Soil Analysis, Thun, Switzerland).

**Soil Characteristics**	
Texture	loamy sand
pH	7.2
**Available nutrient (mg/Kg)**	
Nitrate	26.1
Phosphorus	1.6
Potassium	6.4
Calcium	182.6
Magnesium	10.7
**Reserved macroelements (mg/Kg)**	
Phosphorus	45.4
Potassium	87.5
Calcium	15760.0
Magnesium	368.5
**Other elements (mg/Kg)**	
Boron	0.7
Manganese	382
Copper	15.2
Iron	196

Table S3 Experimental design.

For ITS2 sequencing

**Experiment**	**Soil type**	**Plant genotypes**	**Pots per genotype**	**Plants per pot**
Exp1	NPK_2013	Gifu B-129, *rad1-2*, *rad1-3*, *ram2-3*, *symrk-10*, *cyclops-3*, *ram1-1*, *ram1-2*, *str1-1*, *str1-2*, *ram2-2*	5	5
Exp2	NPK_2016	Gifu B-129, *rad1-2*, *rad1-3*, *ram2-3*, *symrk-10*, *cyclops-3*, *ram1-1*, *ram1-2*, *str1-1*, *str1-2*, *ram2-2*	6	6
Exp3	NPK_2016	Gifu B-129, *ram2-3*, *symrk-10, ram2-2*	6	6

Sand/soil mixture with *R. irregularis* inoculum

**Experiment**	**Soil type**	**Plant genotypes**	**Pots per genotype**	**Plants per pot**
Exp1	Sand/soil	Gifu B-129, *rad1-2*, *rad1-3*, *ram2-3*, *symrk-10*, *cyclops-3*, *ram1-1*, *ram1-2*, *str1-1*, *str1-2*, *ram2-2*	3-4	5
Exp2	Sand/soil	Gifu B-129, *rad1-2*, *rad1-3*, *ram2-3*, *symrk-10*, *cyclops-3*, *ram1-1*, *ram1-2*, *str1-1*, *str1-2*, *ram2-2*	3-4	5

Sand/soil mixture with *Dactylonectria* and *R. irregularis* co-inoculation

**Experiment**	**Soil type**	**Plant genotypes**	**Fungi isolates**	**Pots per genotype**	**Plants per pot**
Exp1	Sand/soil	Gifu B-129, *symrk-10*, *cyclops-3*	107	4	4
Exp2	Sand/soil	Gifu B-129, *symrk-10*, *cyclops-3*	107, 57	4	4
Exp3	Sand/soil	Gifu B-129, *symrk-10*, *cyclops-3*	107, 102	4	4

Table S4 Primers used in this study.

**Primers for genotyping**		
name	Plant Lines	Sequences (5' to 3')
symrk10-F	*symrk-10*	CATTCAACCAAGCTCCACTC
symrk10-R		ATGAGAACCTGGTGCCACTT
rad1-2-F	*rad1-2 (30030576)*	CCGAGGCTCATGCCTAGGTCCACT
rad1-2-R		CCCCAATGGGTTTCCATGCCTATCC
rad1-3-F	*rad1-3 (30052260)*	AGTCGTGGCGCTTTGAATTCGGTG
rad1-3-R		TGAGTGGTTTCGAATCACCAGAGCG
ram1-1-F	*ram1-1 (30002740)*	GCTTGTGCTGAAGCAGTGGCCAAA
ram1-1-R		TGCGGAGGGAGTGTGCTAATTCGG
ram1-2-F	*ram1-2 (30082472)*	TCAATTGCTGGCAGGGCAAGGTTC
ram1-2-R		TGCAGTCCAAGAAAAATGCCACTCA
ram2-2-F	*ram2-2 (30000742)*	GCCTTCGAAGCCGGTGGCATTTTA
ram2-2-R		TCCCAGGCTTGCAAATCATCCCAG
ram2-3-F	*ram2-3 (30002873)*	GCTCACTGATGCTCCCTTCATGGC
ram2-3-R		TGAAAGTATCCGGGTGGGCGTTGA
str-1-F	*str-1 (30001288)*	TGACGTGATCGTTGTGACACTTGAAGC
str-1-R		CGCAGTTCAAGGGCTCACCTTTGC
str-2-F	*str-2 (30055073)*	GCAACAGTTGGGCTTGACCCCCTT
str-2-R		ATGTTCGCCACGAGAGCACTGCAA
P2-R		CCATGGCGGTTCCGTGAATCTTAGG
LORE1a insertion lines: Forward primer combine with P2-R for the amplification of insertions. Amplification from EMS lines was used for sequencing to confirm the mutation.		
**Primers for quantification of fungi**		
Cyl_R		TGTGCTACTACGCAGAGGAA
YT2F		GATGAAGAACGCAGCGAAAT
102_Cyl_R		GTTGCTACTACGCTGAGGTA
gLjUBQ-F		ATGCAGATCTTCGTCAAGACCTTG
gLjUBQ-R		ACCTCCCCTCAGACGAAG
gRiLSU-F		TTCGGGTAATCAGCCTTTCG
gRiLSU-R		TCAGAGATCAGACAGGTAGCC
**Primers for gene expression in real-time PCR**		
qLjBCCP2-F		ACATTCATGGCCCAGGTTTC
qLjBCCP2-R		GTGCTGCTTTTGAAGGTGGA
qLjPT4-F		TCCAAGCGGAGCAAGACAAG
qLjPT4-R		TTCTGTGTGAGGTTCTGGCTGTAG

qLjUbiquitin-F	TTCACCTTGTGCTCCGTCTTC
qLjUbiquitin-R	AACAACAGCACACACAGACAATCC

Table S9 Differences of alpha-diversity (ANOVA on Shannon's H index) and structure (PerMANOVA on Bray-Curtis dissimilarities) of fungal communities in the different plant lines and compartments. All the factors and their interactions were considered. Bold numbers indicate significant *P*-values. Data from experiment 3 was excluded for the analysis because of its incomplete experimental set-up. Glomeromycota OTUs were included.

**Factor**	**Alpha diversity**		**Community structure**	
	Variance explained (%)	*P*-value	Variance explained (%)	*P*-value
**Overall**				
compartment	83%	**2×10^{-16}**	48%	**1×10^{-4}**
experiment	11%	**2×10^{-16}**	13%	**1×10^{-4}**
arb phenotype			3%	**1×10^{-4}**
compartment x experiment			7%	**1×10^{-4}**
compartment x arb phenotype			2%	**1×10^{-4}**
experiment x arb phenotype				
compartment x experiment x arb phenotype				
compartment x experiment x arb phenotype / genotype				
**Root**				
experiment	81%	**2×10^{-16}**	31%	**1×10^{-4}**
arb phenotype			16%	**1×10^{-4}**
experiment x arb phenotype				
experiment x arb phenotype / genotype				
**Rhizosphere**				
experiment	53%	**2×10^{-10}**	43%	**1×10^{-4}**
arb phenotype			2%	**0.004**
experiment x arb phenotype				
experiment x arb phenotype / genotype				
**Bulk soil**				
experiment		0.6		0.1

Table S10 Differences of alpha-diversity (ANOVA on Shannon's H index) and structure (PerMANOVA on Bray-Curtis dissimilarities) of fungal communities in the different plant lines and compartments after exclusion of Glomeromycota OTUs. All the factors and their interactions were considered. Bold numbers indicate significant *P*-values. Data from experiment 3 was excluded for the analysis because of its incomplete experimental set-up. Glomeromycota OTUs were excluded.

Factor	Alpha diversity		Community structure	
	Variance explained (%)	*P*-value	Variance explained (%)	*P*-value
**Overall**				
compartment	84%	**2×10^{-16}**	50%	**1×10^{-4}**
experiment	10%	**2×10^{-16}**	13%	**1×10^{-4}**
arb phenotype			1%	**1×10^{-4}**
compartment x experiment			7%	**1×10^{-4}**
compartment x arb phenotype			1%	**0.001**
experiment x arb phenotype				
compartment x experiment x arb phenotype				
compartment x experiment x arb phenotype / genotype				
**Root**				
experiment	80%	**2×10^{-16}**	38%	**1×10^{-4}**
arb phenotype			5%	**1×10^{-4}**
experiment x arb phenotype				
experiment x arb phenotype / genotype				
**Rhizosphere**				
experiment	53%	**2×10^{-10}**	44%	**1×10^{-4}**
arb phenotype			1%	**0.02**
experiment x arb phenotype				
experiment x arb phenotype / genotype				
**Bulk soil**				
experiment		0.6		0.1

References

Almario J, Jeena G, Wunder J, Langen G, Zuccaro A, Coupland G, Bucher M. 2017. Root-associated fungal microbiota of nonmycorrhizal *Arabis alpina* and its contribution to plant phosphorus nutrition. *Proceedings of the National Academy of Sciences, USA* **114**(44): E9403-E9412.

Gerlach N, Schmitz J, Polatajko A, Schlueter U, Fahnenstich H, Witt S, Fernie AR, Uroic K, Scholz U, Sonnewald U. 2015. An integrated functional approach to dissect systemic responses in maize to arbuscular mycorrhizal symbiosis. *Plant Cell and Environment* **38**(8): 1591-1612.

Gobbato E, Marsh JF, Vernie T, Wang E, Maillet F, Kim J, Miller JB, Sun J, Bano SA, Ratet P, et al. 2012. A GRAS-type transcription factor with a specific function in mycorrhizal signaling. *Current Biology* **22**(23): 2236-2241.

Gutjahr C, Radovanovic D, Geoffroy J, Zhang Q, Siegler H, Chiapello M, Casieri L, An K, An G, Guiderdoni E, et al. 2012. The half-size ABC transporters STR1 and STR2 are indispensable for mycorrhizal arbuscule formation in rice. *Plant Journal* **69**(5): 906-920.

Keymer A, Pimprikar P, Wewer V, Huber C, Brands M, Bucerius SL, Delaux PM, Klingl V, Ropenack-Lahaye EV, Wang TL, et al. 2017. Lipid transfer from plants to arbuscular mycorrhiza fungi. *Elife* **6:**29107.

Love MI, Huber W, Anders S. 2014. Moderated estimation of fold change and dispersion for RNA-seq data with DESeq2. *Genome Biology* **15**(12): 550.

Park HJ, Floss DS, Levesque-Tremblay V, Bravo A, Harrison MJ. 2015. Hyphal branching during arbuscule development requires Reduced Arbuscular Mycorrhiza1. *Plant Physiology* **169**(4): 2774-2788.

Rey T, Bonhomme M, Chatterjee A, Gavrin A, Toulotte J, Yang W, Andre O, Jacquet C, Schornack S. 2017. The *Medicago truncatula* GRAS protein RAD1 supports arbuscular mycorrhiza symbiosis and *Phytophthora palmivora* susceptibility. *Journal of Experimental Botany* **68**(21-22): 5871-5881.

Rich MK, Schorderet M, Bapaume L, Falquet L, Morel P, Vandenbussche M, Reinhardt D. 2015. The Petunia GRAS transcription factor ATA/RAM1 regulates symbiotic gene expression and fungal morphogenesis in arbuscular mycorrhiza. *Plant Physiology* **168**(3): 788-797.

Wang E, Schornack S, Marsh JF, Gobbato E, Schwessinger B, Eastmond P, Schultze M, Kamoun S, Oldroyd GE. 2012. A common signaling process that promotes mycorrhizal and oomycete colonization of plants. *Current Biology* **22**(23): 2242-2246.

Xue L, Cui H, Buer B, Vijayakumar V, Delaux PM, Junkermann S, Bucher M. 2015. Network of GRAS transcription factors involved in the control of arbuscule development in *Lotus japonicus*. *Plant Physiology* **167**(3): 854-871.

Yano K, Yoshida S, Muller J, Singh S, Banba M, Vickers K, Markmann K, White C, Schuller B, Sato S, et al. 2008. CYCLOPS, a mediator of symbiotic intracellular accommodation. *Proceedings of the National Academy of Sciences, USA* **105**(51): 20540-20545.

Yoshida S, Parniske M. 2005. Regulation of plant symbiosis receptor kinase through serine and threonine phosphorylation. *Journal of Biological Chemistry* **280**(10): 9203-9209.

Zhang Q, Blaylock LA, Harrison MJ. 2010. Two *Medicago truncatula* half-ABC transporters are essential for arbuscule development in arbuscular mycorrhizal symbiosis. *The Plant Cell* **22**(5): 1483-1497.
